# Automatic root measurement: a lightweight method for measuring pea root length

**DOI:** 10.1186/s13007-025-01479-1

**Published:** 2025-12-08

**Authors:** Haoyu Jiang, Chenhan Hu, Luxu Tian, Tengfei Liu, Weili Sun, Xiuqing Fu, Chenhao Jin, Bo Zhang, Fei Hu

**Affiliations:** 1 College of Artificial Intelligence, Nanjing Agricultural University, Nanjing, 210031 China; 2 Glasgow College, University of Electronic Science and Technology of China, Chengdu, 611731 China; 3 College of Engineering, Nanjing Agricultural University, Nanjing, 210031 China

**Keywords:** Pea seeds, Root length measurement, Cost-effective deployment, Instance segmentation, Stress-resistant crop breeding

## Abstract

**Background:**

With the intensification of global climate change, extreme weather events have become increasingly frequent, severely impacting the growth cycles and yield stability of crops. Against this backdrop, cultivating new crop varieties with high stress resistance has become a core task for achieving sustainable agriculture and ensuring food security. Root length, as a critical phenotypic trait that reflects a plant’s ability to absorb water and nutrients, is closely related to the crop’s capacity to withstand adversities, such as drought, high temperatures and salinisation. However, root length measurement technology remains a significant bottleneck in plant science research. Traditional manual methods are inefficient and prone to human-induced variability (e.g. subjective standard discrepancies, operational errors, and potential contamination or damage to seeds). Meanwhile, existing automated measurement models face challenges in large-scale practical applications due to their high deployment costs.

**Results:**

This study developed a seed germination image acquisition system and constructed a pea root dataset. Based on the YOLOv8-Seg-n instance segmentation model, a lightweight automatic root measurement (ARM) model was then developed using feature distillation, structured pruning techniques, and a series of post-processing procedures for root length calculation. Experimental results demonstrated that the ARM model had only 1.81 M parameters, with 8.3 GFLOPs and a weight file size of 4.2 MB, and achieved 70.4 FPS. It realised outstanding performance with mAP@0.5 and AP_root_ scores of 90.3% and 81.2%, respectively, showing a high consistency with manual measurement results (R² = 0.993). Compared to existing models, the ARM model significantly reduces parameter scale and computational complexity, making it more accommodating to device performance and computational requirements while also decreasing the workload associated with root sample processing. Furthermore, the application of the ARM model in a 72-hour full time-series analysis of pea root length under drought conditions validated its potential for practical use in real-world scenarios.

**Conclusions:**

The ARM model offers an efficient and cost-effective technological solution for high-throughput root length measurement in peas. It achieves a favorable balance between accuracy, speed, and computational resource requirements, demonstrating broad application potential in agricultural production and breeding research. The model offers critical technical support for ensuring food security and enhancing crop stress resistance.

**Graphical abstract:**

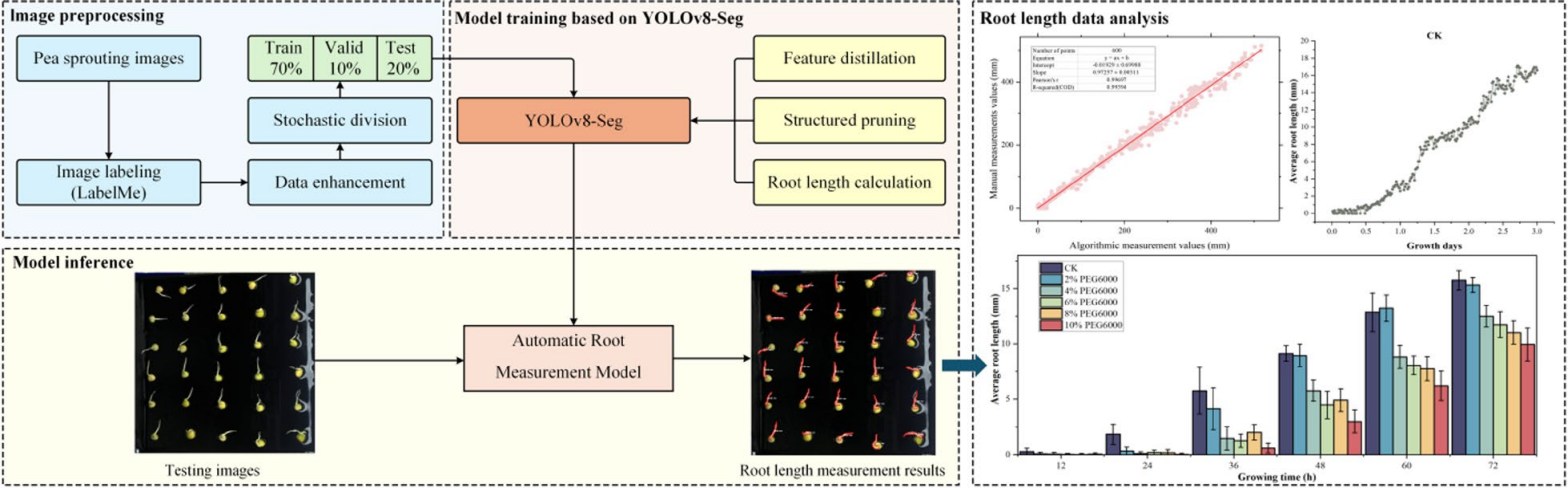

**Supplementary Information:**

The online version contains supplementary material available at 10.1186/s13007-025-01479-1.

## Introduction

The plant root system is the core organ for crop–soil interaction, which is not only responsible for water and nutrient absorption but is also playing crucial roles in supporting plant stability, regulating stress responses and interacting with soil microorganisms. The developmental status of the root system directly determines the efficiency of crop water and nutrient utilisation, ultimately influencing crop growth cycles and yields [1, 2]. Analyses of root morphology, distribution and length hold significant value across various fields, including breeding optimisation, transpiration estimation, precision irrigation, food security and climate change [3–6]. Such studies not only provide scientific evidence for improving crop resource utilisation efficiency but also offer reliable approaches for enhancing crop stress resistance.

The root system demonstrates remarkable plasticity, with its morphology and functions being shaped by genetic factors and environmental conditions, particularly under stresses, such as drought, salinity and nutrient deficiency [7–9]. Environmental stresses significantly influence root development and functionality by altering soil water availability, nutrient accessibility and ionic balance [10, 11]. For instance, drought stress often drives roots to grow deeper into the soil to access water, which may suppress lateral root growth and reduce total root biomass [12–14]. Salinity stress disrupts soil osmotic potential and introduces ionic toxicity, impairing root apical meristem activity and leading to inhibited root growth or tissue damage [7, 15, 16]. Nutrient deficiencies, such as insufficient nitrogen or phosphorus, typically promote root elongation or the formation of additional root hairs, enhancing the plant’s ability to absorb limited nutrients [17, 18]. This plasticity of the root system under environmental regulation is a key phenotypic trait for assessing crop stress resistance. By analysing the dynamic responses of roots to stress conditions, researchers can uncover the adaptive mechanisms of crops, providing a scientific foundation for the precise breeding of stress-resistant traits and the improvement of germplasm.

Considering the unique biological characteristics and significant agricultural value of pea (*Pisum sativum*), this study selects pea as the research subject. As a globally important legume crop, pea not only occupies a crucial position in food production and feed supply but also makes a significant contribution to the sustainable development of agricultural ecosystems through its symbiosis with nitrogen-fixing microorganisms [19]. Under stress conditions, the root system of pea often exhibits notable morphological adjustments. Monitoring root length and growth rate can help elucidate the mechanisms by which the root system responds to varying growth environments, water availability and nutrient supply. This analysis is not only key to improving crop adaptability and productivity under diverse environmental conditions but also provides critical scientific evidence for breeding stress-resistant varieties [20].

However, traditional methods for measuring root length, such as profile wall measurement, block excavation and soil core analysis, require extensive labour, consume considerable time and disrupt root structures, causing morphological alterations that lead to inaccurate measurements and biased conclusions [21–23]. Additionally, these methods heavily depend on the subjective judgment of operators, limiting the throughput and accuracy of root length measurements, as well as the reproducibility and reliability of results [24]– [25]. Modern root imaging techniques, such as X-CT, NMRI and radioactive isotope tracing, have developed rapidly [26], but their high cost, operational complexity and dependence on laboratory environments make them challenging for widespread application [27]. Therefore, developing a high-accuracy, automated and high-throughput root length measurement method is critical for addressing these challenges, improving research efficiency and enhancing the reliability of results and holds significant research value.

**Fig. 1:**
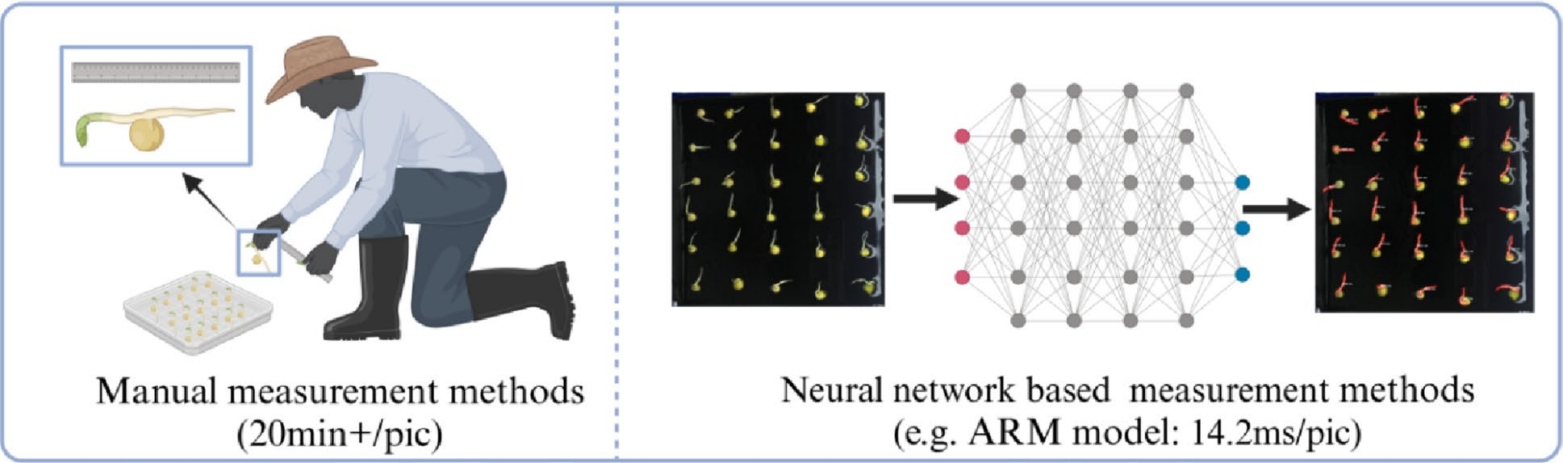
Comparison of the efficiencies of manual and neural network-based methods for root length measurement

Computer vision technology, capable of processing large-scale image data in parallel and extracting features effectively, enables nondestructive, full time-series, high-throughput and high-accuracy phenotypic detection of root length throughout the seed germination process [28–30]. The current mainstream methods can be divided into two major technical paradigms: Transformer-based and Convolutional Neural Networks (CNNs) based approaches. Transformer-based architectures model long-range dependencies through global self-attention mechanisms. Representative examples include ViT [31], DETR [32], and Mask2Former [33]. While these methods effectively capture global contextual information in images, their dense computational characteristics limit training convergence speed and impose high demands on computational resources and memory. CNN-based frameworks, on the other hand, are primarily divided into two-stage and single-stage architectures. The two-stage R-CNN series [34]– [35] first generates candidate regions using a region proposal network (RPN), then extracts features through CNNs, and finally classifies and regresses bounding boxes using a classifier. While this architecture achieves good detection accuracy, it significantly increases computational overhead due to redundant computations in the region proposal and feature extraction stages. The single-stage You Only Look Once (YOLO) series [36–42] adopts a global grid encoding strategy, dividing the image into dense prediction units that directly regress bounding box parameters and classification probabilities. By eliminating redundant computation stages through an end-to-end architecture, it achieves significant advantages in computational efficiency. Notably, the collaborative application of multi-scale feature fusion and Cross-Stage Partial Networks (CSP) technology, along with dynamic loss function optimisation, effectively enhances the accuracy of feature encoding. Leveraging this series of design features, the YOLO architecture has been effectively applied to a wide range of agricultural tasks, demonstrating high detection accuracy and robustness (Table 1).

**Table 1:** Summary of existing research on YOLO for agricultural applications

Model	References	Research focus
YOLOv8	Tian et al. [43]	Tomato seed germination vigor phenotyping
	Sun et al. [44]	Pear pollen phenotypic analysis
YOLOv9	Lu et al. [45]	Multi-crop weed/crop detection
	Liang et al. [46]	Soybean seedling and weed discrimination
YOLOv10	Yuan et al. [47]	Agricultural pest detection
YOLOv11	Xiao et al. [48]	Crop detection in remote sensing imagery
	Teng et al. [49]	Rice disease detection
	Eliwa et al. [50]	Multi-crop plant disease classification
	Zhang et al. [51]	Peanut leaf spot disease detection and severity quantification
YOLOv12	Yang et al. [52]	Multi-crop weed detection
	Sapkota et al. [53]	Apple detection

Recently, many scholars have begun exploring the application of CNNs in root analysis [54]. For instance, Shen et al. [55] designed an automatic cotton root image segmentation method based on the DeepLabv3 convolutional neural network architecture, significantly reducing the time required for measurement. Khoroshevsky et al. [56] proposed a CNN-based framework capable of accurately estimating total root length, average root diameter and white root percentage directly from MR images without requiring root segmentation. Han et al. [57] developed RootPainter, a U-Net-based software that enables the precise calculation of root density and root length density in different soil profile images. Peters et al. [58] introduced RootDetector, a neural network architecture for detecting bud and root length in micro-root nodule images under field conditions, achieving accuracy comparable to manual measurements. Betegón-Putze et al. [59] developed MyROOT software, which integrates a bottom–up root tracking method with a hypocotyl detection algorithm to measure the root length of *Arabidopsis thaliana*. Wang et al. [60] proposed SegRoot, a fully automated method based on convolutional neural networks that effectively segments root system images from complex soil backgrounds, producing predictions highly correlated with manual detection (R² = 0.9791). Huang et al. [61] enhanced the OCRNet model by incorporating a global attention mechanism (GAM) module, improving the model’s focus on root targets and achieving an accuracy of 0.9866 in high-resolution micro-root canal image segmentation. These studies demonstrated that integrating plant root system research with convolutional neural networks can not only achieve results comparable to manual measurements but can also significantly improve computational efficiency, driving the field of root detection towards greater automation and precision (Fig. 1).

Although root measurement technologies based on deep learning have achieved significant progress in accuracy, their high precision often relies on complex network architectures. This dependence on high-performance computing devices poses high requirements for equipment deployment in practical agricultural applications, limiting large-scale adoption in agricultural production. Currently, research on model size optimisation and computational speed improvement remains insufficient. Complex models struggle to balance efficiency and hardware compatibility, thereby affecting practical application benefits. In addition, achieving full time-series dynamic monitoring and real-time feedback of root growth faces numerous technical bottlenecks. Not only is a breakthrough in the efficiency of real-time algorithms for processing dynamic data required, but specialised equipment that integrates environmental adaptability, refined human–computer interaction design, environmental parameter regulation and data collection management optimisation must also be developed urgently to meet the systemic requirements of full time-series monitoring. To promote the large-scale application of this technology in agricultural production, it is essential to address the aforementioned challenges related to device performance, algorithm efficiency, and adaptability to application scenarios. In terms of equipment development, the focus should be on designing monitoring systems with full time-series sequence capability and high throughput. For algorithm optimisation, emphasis should be placed on enhancing the model’s lightweight architecture and real-time responsiveness. Additionally, a comprehensive solution with low deployment costs and flexible adaptability should be developed, tailored to the practical needs of agricultural production, to facilitate the transition of the technology from laboratory research to real-world applications. The main contributions of this study are as follows:

1. Development of full time-series monitoring equipment: A seed germination image acquisition system integrating environmental control and image collection was developed, enabling real-time analysis of phenotypic parameters throughout the entire seed germination process.
2. Construction of datasets: Two datasets for crop root growth analysis were developed: a pea germination dataset containing 2,400 images and a drought stress dataset containing 2,592 images.
3. Model optimisation for resource-constrained scenarios: The baseline model was optimised using knowledge distillation and model pruning techniques to improve parameter utilisation efficiency and accelerate inference speed. Additionally, a series of root length calculation postprocessing procedure, including contour extraction, perimeter calculation and scale conversion, was designed to construct an ARM model for root length measurement. This model provides an efficient and economical solution, offering an alternative to traditional manual measurement methods.
4. Validation of model application: The ARM model’s performance in full time-series, high-precision and high-throughput measurement of pea root length was validated using Macrogol 6000 (PEG-6000 solution) for simulating drought environments, demonstrating its applicability in real-world scenarios.

## Materials and methods

### Acquisition system for seed germination images

**Fig. 2:**
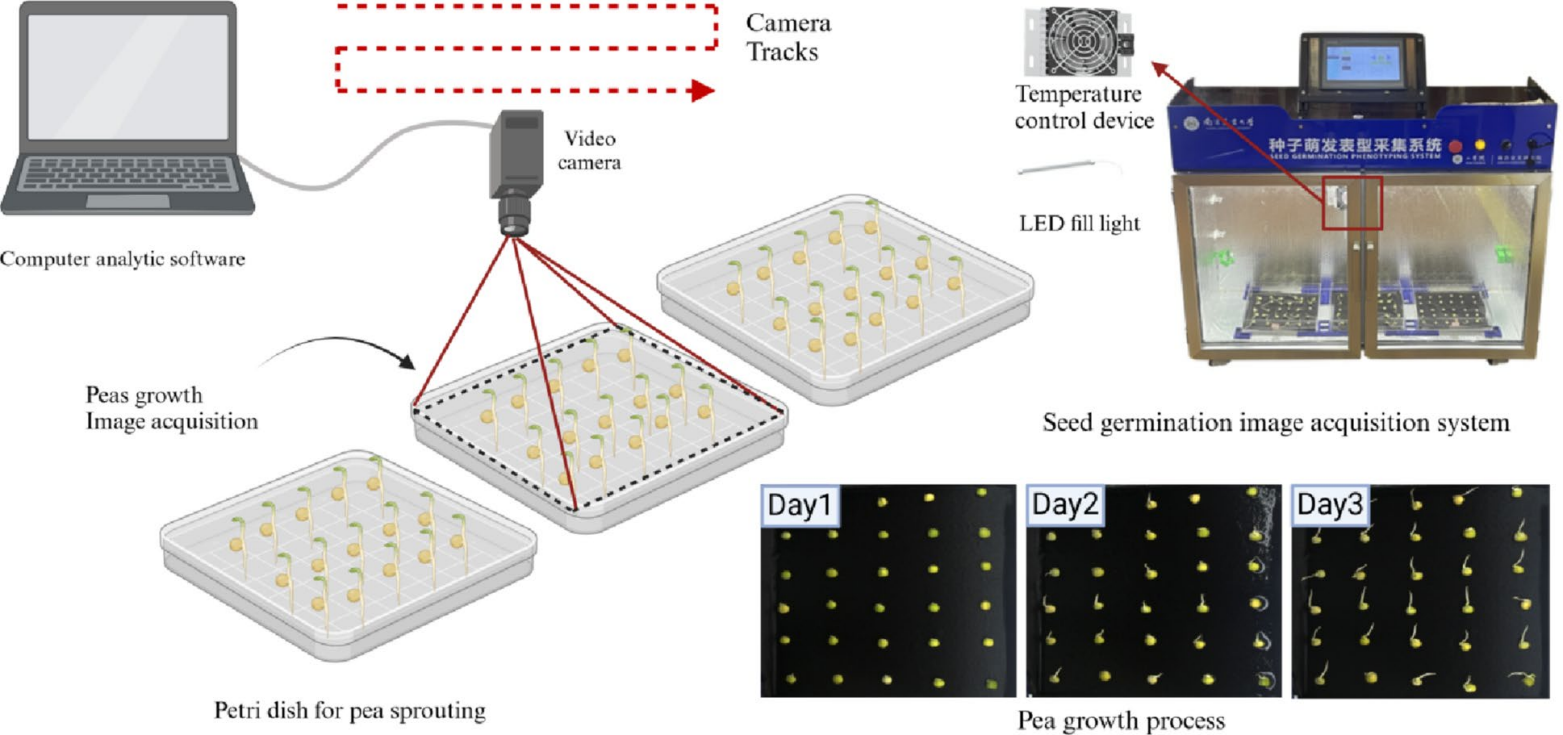
Seed germination image acquisition system

To enable full time-series sequence acquisition of pea germination phenotypic images, this study developed a seed germination image acquisition system, as illustrated in Fig. 2. This system integrates environmental control and image acquisition into an intelligent agricultural device. Through a human–machine interaction system, system parameters can be configured to achieve the full time-series and high-throughput acquisition of pea germination images. The device uses a rail-mounted camera to capture images of three trays of crops, with the shooting path indicated by the red arrows in the figure.

The device is equipped with electric heating and thermocouple temperature measurement technology to meet the environmental needs for different crop germination processes, offering a temperature range that is adjustable from 10 °C to 50 °C with a control precision of 0.1 °C. It can achieve a 5 °C temperature adjustment within 30 min. Humidity is regulated by a humidifier and measured by using resistive sensors. It can be increased by 10% within 30 min, with control maintained between 30% and 70% relative humidity.

Image acquisition was performed using a high-resolution Hikvision industrial camera (MV-CS060-10GC) and a Computar lens (MVL-HF1228M-6MPE), offering a resolution of 3072 × 2048 pixels and a focal length of 12 mm. The camera captures 19.1 frames per second, with an axial movement error of only ± 1 mm, ensuring high accuracy in image acquisition. The device is equipped with a four-port gigabit switch, with a software response time of 100 ms and 1 GB of memory. It operates at a total power of 800 W, using a 220 V 50 Hz power supply, and connects via a TCP–IP interface. It supports fixed-point shooting and remote control, as well as image cropping and classification storage, and is compatible with JPG and PNG formats.

The custom-designed PC software allows users to set parameters, such as shooting intervals, contrast, and image size, ensuring the precise acquisition of images of different seed germination stages and storing images at designated locations. This system provides real-time image capture throughout seed germination, offering a wealth of high-quality data to support model training.

### Data acquisition process

**Fig. 3:**
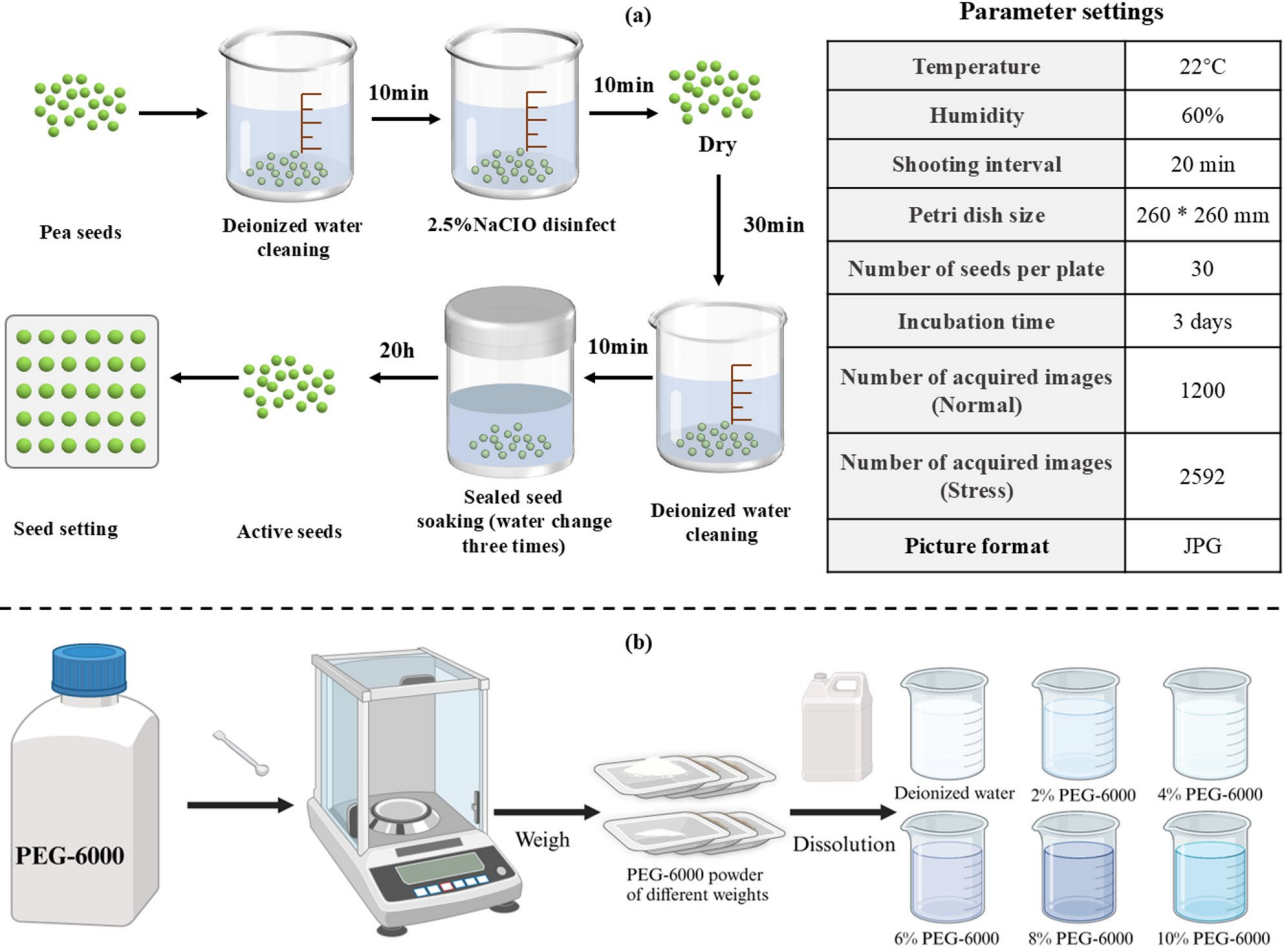
Flowchart of the pea germination experiment. (a) Flow of pea germination test. (b) Preparation process of PEG-6000 solution with difference concentrations

In this study, 180 uniform and plump seeds of the ‘Zhonghua No. 6’ pea genotype were selected for the seed germination experiment. The process is shown in Fig. 3a. Prior to the start of the experiment, the seeds were first soaked in deionized water for 10 min for cleaning, then subjected to surface disinfection with a 2.5% sodium hypochlorite solution. After being air-dried for 30 min, the seeds underwent a second round of cleaning. The cleaned seeds were then soaked in a sealed beaker for 20 h for activation, with water being changed three times during this period. The activated seeds were subsequently placed in petri dishes made from acrylic glass, each with dimensions of 260 mm × 260 mm. Each petri dish held 30 seeds, and each experiment lasted for three days, enabling the simultaneous collection of germination images for 90 pea seeds. During the experiment, the temperature was maintained at 22 °C and the humidity was set at 60%, with germination images captured every 20 min. The experiment was repeated twice. After screening, a total of 1200 images were collected during the experiment. All images were saved in .jpg format.

To validate the applicability of the ARM model in real-world scenarios, this study utilized 360 pea seeds of the ‘Zhonghua No. 6’ genotype as experimental subjects. Drought stress experiments were conducted under various conditions, enabling full time-series sequence measurements of pea root length, and systematically analyzed the effects of adverse conditions on the growth of pea root length. A PEG-6000 solution was used to inhibit water absorption by the pea seeds, simulating a drought environment. Each experiment lasted for three days. Under the same environmental settings, six different concentration treatments were applied: a control check (CK) with deionized water and drought conditions simulated by 2%, 4%, 6%, 8%, and 10% PEG-6000 solutions. Two replicates were set for each concentration to reduce error, and the preparation of the PEG-6000 solutions with different concentrations is shown in Fig. 3b.

During the experiment, the mass of the solution in each petri dish was weighed daily, and the solution was refreshed by preparing new PEG-6000 solutions at the appropriate concentrations to ensure that the solution concentration and quality remained consistent. A total of 2592 images were collected during the drought stress experiment. These images were used for the subsequent full time-series analysis of pea root length.

### Data preprocessing

#### Data annotation

In this study, the Labelme tool [62] was employed to annotate the contours of pea root and seed regions. This joint annotation of seeds and roots enhances the model’s ability to extract and localise root features while minimising background interference. Annotating only the root regions could lead to confusion in the boundary areas between roots and seeds, resulting in blurred segmentation boundaries.

#### Data augmentation

**Fig. 4:**
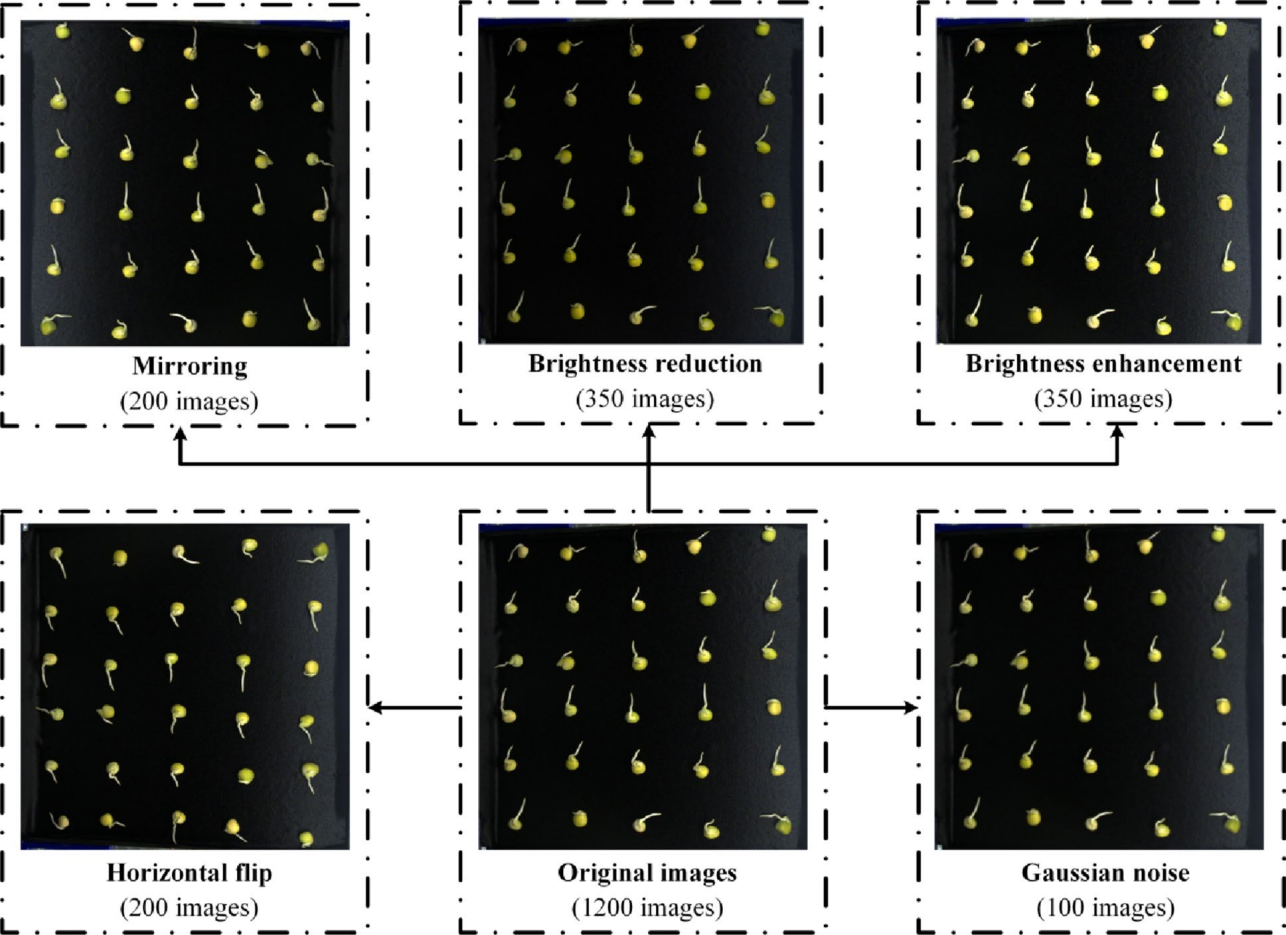
Data augmentation

Given the nonlinear growth characteristics of pea root systems, as well as interference from water surface reflections and noise during image acquisition, the complexity and time cost of root length measurement remarkably increased. Three data augmentation techniques-brightness adjustment, flipping, and Gaussian noise-were applied to enhance the images and their corresponding labels and thus effectively tackle the above challenges and improve the robustness of the proposed method: (1) Brightness adjustment was used to improve the model’s recognition ability under varying lighting conditions, addressing brightness fluctuations that occur in real-world applications. (2) Image mirroring and horizontal flipping were applied to simulate different orientations of pea root growth, enhancing the model’s robustness and generalisation ability in handling the diversity of root growth and spatial transformations. (3) Gaussian noise was added to simulate disturbances encountered in real-world imaging (e.g., sensor noise caused by poor lighting conditions or high temperatures), thereby strengthening the model’s resistance to noise interference. The specific data augmentation process is illustrated in Fig. 4.

#### Dataset partitioning

The final dataset used in this study consisted of 2,400 images, including 1,200 original images and 1,200 augmented images. Of these, 70% (1,680 images) were allocated to the training set to support the training process of the ARM model. The validation set accounted for 10% (240 images) and was used to select the optimal parameters of the network structure and prevent overfitting. The remaining 20% (480 images) comprised the test set, which was used to evaluate the model’s performance and simulate its behaviour when handling unseen data.

### ARM model design

This subsection presents the construction method of the ARM model. First, the YOLOv8-Seg-n instance segmentation model structure was optimized using feature distillation and structured pruning techniques, enabling precise segmentation of pea root regions while reducing the model’s parameter complexity. Subsequently, post-processing operations, including contour extraction, perimeter calculation, and scale conversion, were applied to the segmentation results. This process culminated in the development of the ARM model, capable of accurately measuring the root length of each pea.

#### YOLOv8-Seg-n instance segmentation model

The goal of instance segmentation algorithms is to perform pixel-level segmentation for each individual object in an image [63]. In contrast to semantic segmentation algorithms, instance segmentation algorithms not only require recognising different semantic categories but also distinguishing between different instances within the same category. This task primarily involves two objectives: (1) performing the fine-grained segmentation of each object instance’s contour; (2) determining the category of the segmented region and distinguishing different instances within the same category. The model must understand the shape, size, boundaries, and interrelationships of objects in an image. The main challenge of instance segmentation is how to integrate boundary and category information accurately to distinguish each entity at the pixel level.

**Fig. 5:**
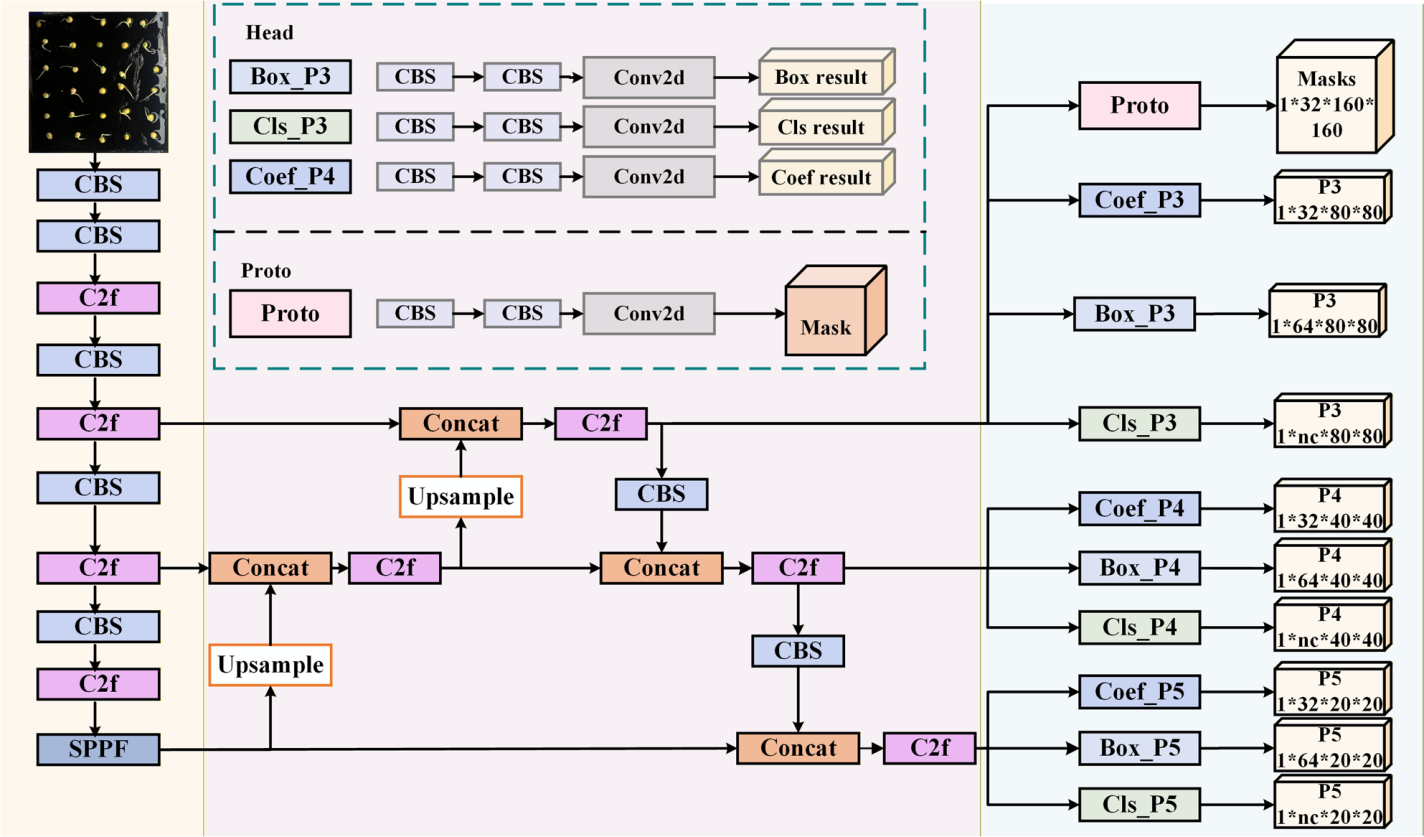
Structure of the YOLOv8-Seg-n instance segmentation model

The YOLOv8-Seg-n instance segmentation model was adopted as the baseline to achieve the precise segmentation of pea root and seed regions. This model decomposes the instance segmentation task into two parallel subtasks, effectively balancing model accuracy and processing speed. Its structure is shown in Fig. 5.

The specific operational flow of the YOLOv8-Seg-n model for instance segmentation is as follows: First, the backbone network of the YOLOv8-Seg-n architecture is used for feature extraction from the input image. Subsequently, a feature pyramid network is employed to synthesize image features from different levels, enhancing the model’s adaptability to scale variations. Based on P3-level feature extraction, the model introduces a parallel mask branch, which works simultaneously with the traditional object detection branch. The mask branch is specifically responsible for generating a set of prototype masks, whereas the object detection branch outputs the class and bounding box information of the detected objects. Additionally, the object detection branch outputs a set of mask coefficients, which indicate each prototype mask’s contribution to the final instance segmentation mask, with values ranging from 1 to − 1.

**Fig. 6:**
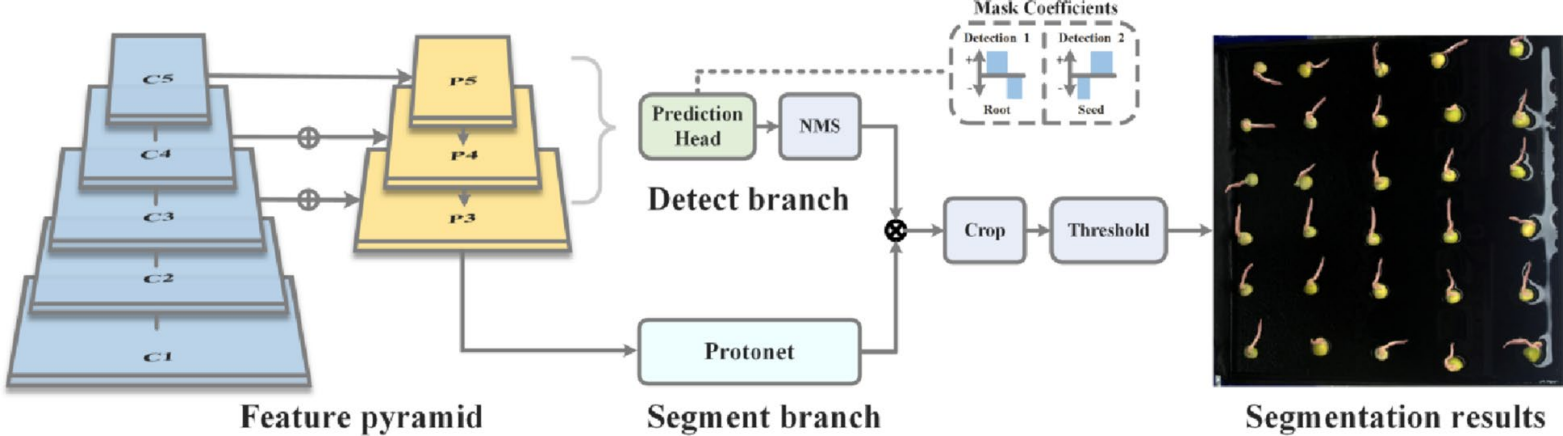
YOLOv8-Seg-n instance segmentation model mask segmentation process

For each target entity in the image, the system calculates the final instance segmentation mask by performing a weighted sum, combining the mask coefficients with their corresponding prototype masks. The mask segmentation process is illustrated in Fig. 6. This method not only allows for the clear delineation of each target’s boundaries within the image, but it also provides detailed class labels and confidence scores for each target. Instance segmentation technology offers positional information and precise shape details of the target instances, adding rich dimensions for a deep understanding of the image content.

#### Lightweight design of the model

Instance segmentation algorithms, due to their ability to provide precise contour information of target instances, greatly facilitate the efficient extraction and analysis of crop phenotypic parameters. However, when applying these algorithms in real-world agricultural production, factors beyond high accuracy must be considered. Such factors include computational efficiency, real-time performance, and deployment costs. Therefore, the model based on YOLOv8-Seg-n was further optimised to enhance these aspects while maintaining accuracy, reducing model complexity, and minimising the demand for computational resources.

1. Feature distillation
  Considering the complexity of the pea root instance segmentation task, achieving precise segmentation for accurate length measurement often requires employing sophisticated network architectures to extract fine-grained features. However, such complex architectures inevitably increase inference time and deployment costs, thereby limiting their feasibility in practical applications.
  Feature distillation techniques [64] can alleviate these challenges to some extent: first, a complex teacher model is trained to effectively learn and express the intricate features of pea roots. Subsequently, the lightweight student model is aligned with the teacher model across multiple feature layers, transferring the teacher model’s feature representation capabilities to the student model. This process enables the student model to more accurately capture the intrinsic structures and patterns of the data, maintaining high precision while reducing model complexity. Additionally, it enhances the model’s generalisation ability when dealing with new data distributions.The schematic of the feature distillation method is illustrated in Fig. 7.
    **Fig. 7:**
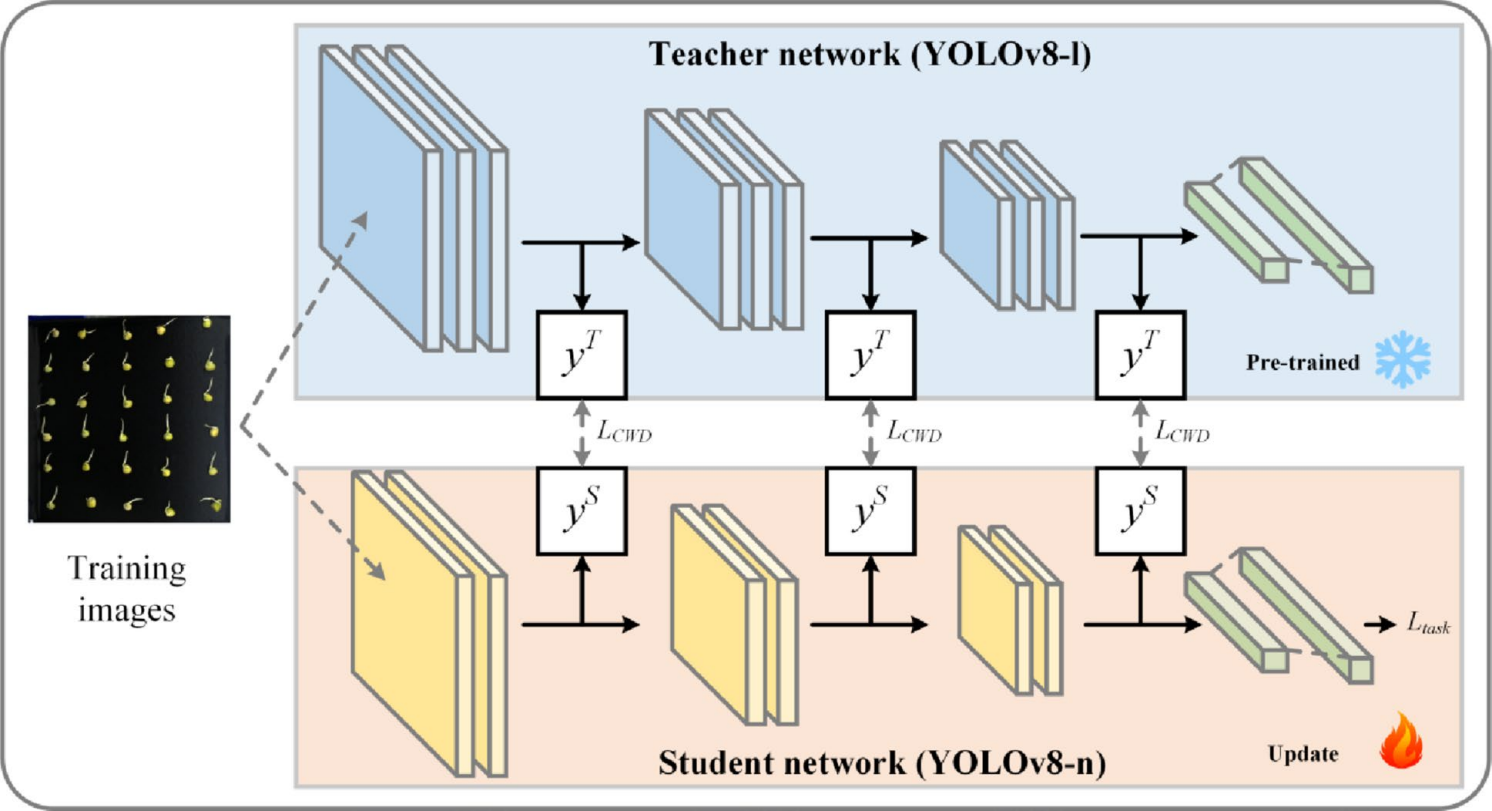
Schematic of the feature distillation method
    When implementing feature distillation, specific intermediate layers from the teacher and student models are selected as the hint and guidance layers, respectively, as shown in Eqs. 1 and 2. Here, $\:N$ represents the feature map size, $\:N\:$= $\:W$ × $\:H$, where $\:W$ and $\:H$ denote the width and height, respectively. The output from the hint layer of the teacher model serves as the learning target for the corresponding guidance layer in the student model, enabling deep knowledge transfer. When processing the pea germination image $\:\varvec{X}$ for segmentation, the teacher model $\:{\varvec{F}}_{t}$ and student model $\:{\varvec{F}}_{S}$ extract features $\:{y}^{T}$ and $\:{y}^{S}$ from their respective hint and guidance layers, with channel numbers $\:{C}_{T}$ and $\:{C}_{S}$, respectively. Considering the structural differences between the teacher and student models, the initial number of channels in the hint and guidance layers may differ. To address this difference, a 1 × 1 convolutional layer is added after the student model’s guidance layer to adjust the number of channels, ensuring that it matches the output from the teacher model’s hint layer. With this design, the student model can effectively learn key feature representations from the teacher model even when structural differences exist, thereby optimising its segmentation performance.
    1 $$\:{y}^{T}={\varvec{F}}_{t}\left(\varvec{X}\right)\in\:{\mathbb{R}}^{{C}_{T}\times\:N},$$
    2 $$\:{y}^{S}={\varvec{F}}_{S}\left(\varvec{X}\right)\in\:{\mathbb{R}}^{{C}_{S}\times\:N}.$$
    Equations 3 and 4 define the calculation method for channel distillation loss between the hint and guidance layers, using Kullback–Leibler (KL) divergence to quantify the difference in channel distribution between the teacher and student networks. The asymmetric nature of KL divergence provides ideal conditions for knowledge distillation, allowing the student network to prioritise absorbing highly representative activation patterns from the teacher network. These representative activations typically correspond to salient or foreground objects in the image while paying little attention to background regions or unimportant features. By quantifying the difference in activation probability distributions on corresponding channels between the teacher and student networks, the student network learns how to match the activation distribution that highlights the importance of different features or categories in the input data, thereby improving its learning efficiency and prediction accuracy. Furthermore, by focusing on the most informative parts of the input data, wherein the teacher network places the greatest emphasis on its activations, the student model’s feature extraction capabilities are further enhanced.
    3 $$\:{Loss}_{CWD}=\varphi\:\left(\phi\:\left({y}^{T}\right),\phi\:\left({y}^{S}\right)\right)= \varphi\:\left(\phi\:\left({y}_{c}^{T}\right), \phi\:\left({y}_{c}^{S}\right)\right),$$
    4 $$\:\varphi\:\left({y}^{T},{y}^{S}\right)=\frac{{\mathcal{T}}^{2}}{C}{\sum\:}_{c=1}^{C}\:{\sum\:}_{i=1}^{W\cdot\:H}\:\phi\:\left({y}_{c,i}^{T}\right)\cdot\:\text{l}\text{o}\text{g}\left[\frac{\phi\:\left({y}_{c,i}^{T}\right)}{\phi\:\left({y}_{c,i}^{S}\right)}\right].$$
    In Eq. 5, $\:\phi\:(\cdot\:)$ is used to convert activation values into probability distributions, reducing the magnitude differences between the more complex teacher network (a large-scale network) and the simpler student network (a compact network) through Softmax normalization. This approach enhances the effectiveness of knowledge transfer, as shown in Eq. 4, where $\:C$ represents the channel index ($\:c$ = 1, 2, ……$\:C$), and $\:i$ denotes the spatial position in the feature map of the indexed channel. $\:\mathcal{T}$ represents the temperature coefficient, which controls the distribution tendency across spatial regions in the probability distribution.
    5 $$\:\phi\:\left({y}_{c}\right)=\frac{\text{e}\text{x}\text{p}\left(\frac{{y}_{c,i}}{\mathcal{T}}\right)}{{\sum\:}_{i=1}^{W\cdot\:H}\:\text{e}\text{x}\text{p}\left(\frac{{y}_{c,i}}{\mathcal{T}}\right)}.$$
        
    The total loss for training the student model during knowledge distillation ($\:{Loss}_{Distillation}$) is defined in Eq. 6, which consists of two components: $\:{Loss}_{Segment}$ and $\:{Loss}_{CWD}$. $\:{Loss}_{Segment}$ represents the student model’s loss during the instance segmentation task, whereas $\:\alpha\:$ is an adjustable hyperparameter, set to 1 in this study.
    6 $$\:{Loss}_{Distillation}={Loss}_{Segment}+\alpha\:{Loss}_{CWD}.$$
2. Structured pruning strategy
  To further reduce the deployment cost of the model, structured pruning was applied to the distilled student model, YOLOv8-Seg-n. By performing sparse training on the network model, channels that have minimal effects on performance are pruned, followed by fine-tuning to recover accuracy. The goal is to reduce the model’s size and computational complexity by removing certain parameters (“pruning”) while maintaining performance as much as possible [65].
  **Fig. 8:**
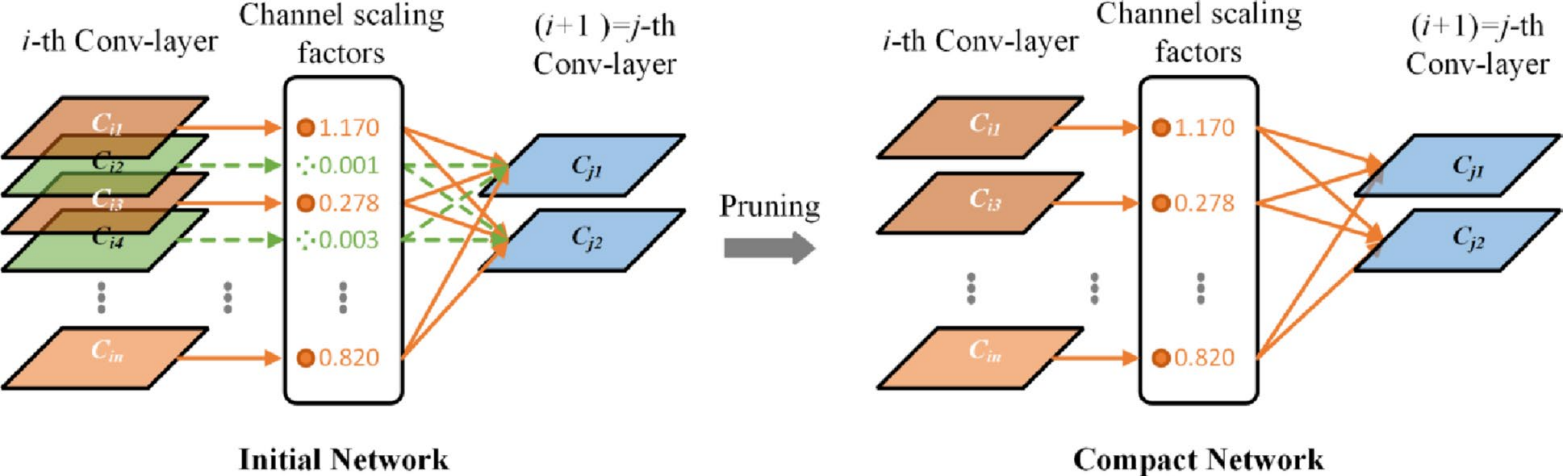
Effect of structured pruning
  The effects of structured pruning are shown in Fig. 8. In the model’s network structure, batch normalization (BN) layers are utilized to mitigate internal covariate shift, reduce the sensitivity of the network to initial parameter values, and effectively enhance the convergence speed of the model. In BN layers, the activation value of each channel is directly proportional to a learnable scaling factor$\:\:\gamma\:$, whose magnitude determines the importance of channel information. Since the activation values output by BN layers typically follow a normal distribution, with most values not approaching zero, L1 regularization ($\:{\left|\:\right|}_{1}$) is introduced in this study to promote sparsity by reducing the values of the importance quantification metric for channels.
  To achieve a highly sparse model while maintaining segmentation accuracy, sparse training requires analyzing the weight distribution of BN layers and the pruning ratio to determine an appropriate pruning threshold. This threshold identifies which parameters should be pruned, thereby balancing the degree of model lightweighting with its feature extraction capability. A higher pruning ratio compresses the model further by removing more channels, but this may come at the cost of reduced accuracy. The BN layer is defined in Eq. 7, where $\:{z}_{\text{out}}$ and $\:{z}_{\text{in}}$ represent the input and output feature data of the BN layer, respectively. $\:{\mu\:}_{B}$ and $\:{\sigma\:}_{B}$ represent the mean and standard deviation of the input data for batch $\:B$, $\:\beta\:$ represents the bias, and $\:\epsilon$ is a small constant used to prevent division by zero in the denominator. The loss function is defined in Eqs. 8 and 9. $\:\:g\left(\gamma\:\right)$ denotes the L1 regularization term, and$\:\:\lambda\:$ is a penalty factor used to counterbalance the regularization loss component.
  7 $$\:{z}_{\text{out}}=\gamma\:\frac{{z}_{\text{in}}-{\mu\:}_{B}}{\sqrt{{\sigma\:}_{B}^{2}+\epsilon}}+\beta\:,$$
  8 $$\:{Loss}_{Pruning\:}={Loss}_{Segment}+\lambda\:{\sum\:}_{\gamma\:\in\:{\Gamma\:}}\:g\left(\gamma\:\right),$$
  9 $$\:g\left(\gamma\:\right)={\left|\gamma\:\right|}_{1}.$$
  This study employs an iterative structured pruning method to reduce the model’s parameters efficiently and thus achieve optimal pruning results. In each pruning iteration, an appropriate threshold is set to prune a portion of the parameters, ensuring that the model’s performance remains unaffected. After each pruning step, the model’s parameters are fine-tuned over multiple rounds by using the training data. By repeating this process, the model’s size gradually reduces, effectively decreasing the number of parameters without sacrificing segmentation accuracy, ultimately resulting in a pruned, sparse network. The detailed structured pruning process is shown in Fig. 9.
  **Fig. 9:**
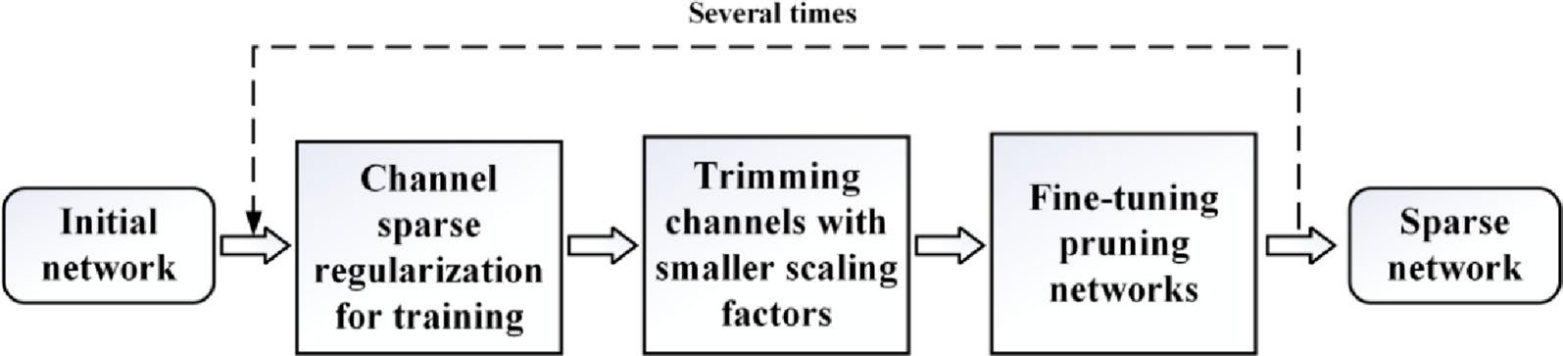
Flowchart of structured pruning

#### Method for the calculation of pea root length based on instance segmentation results

**Fig. 10:**
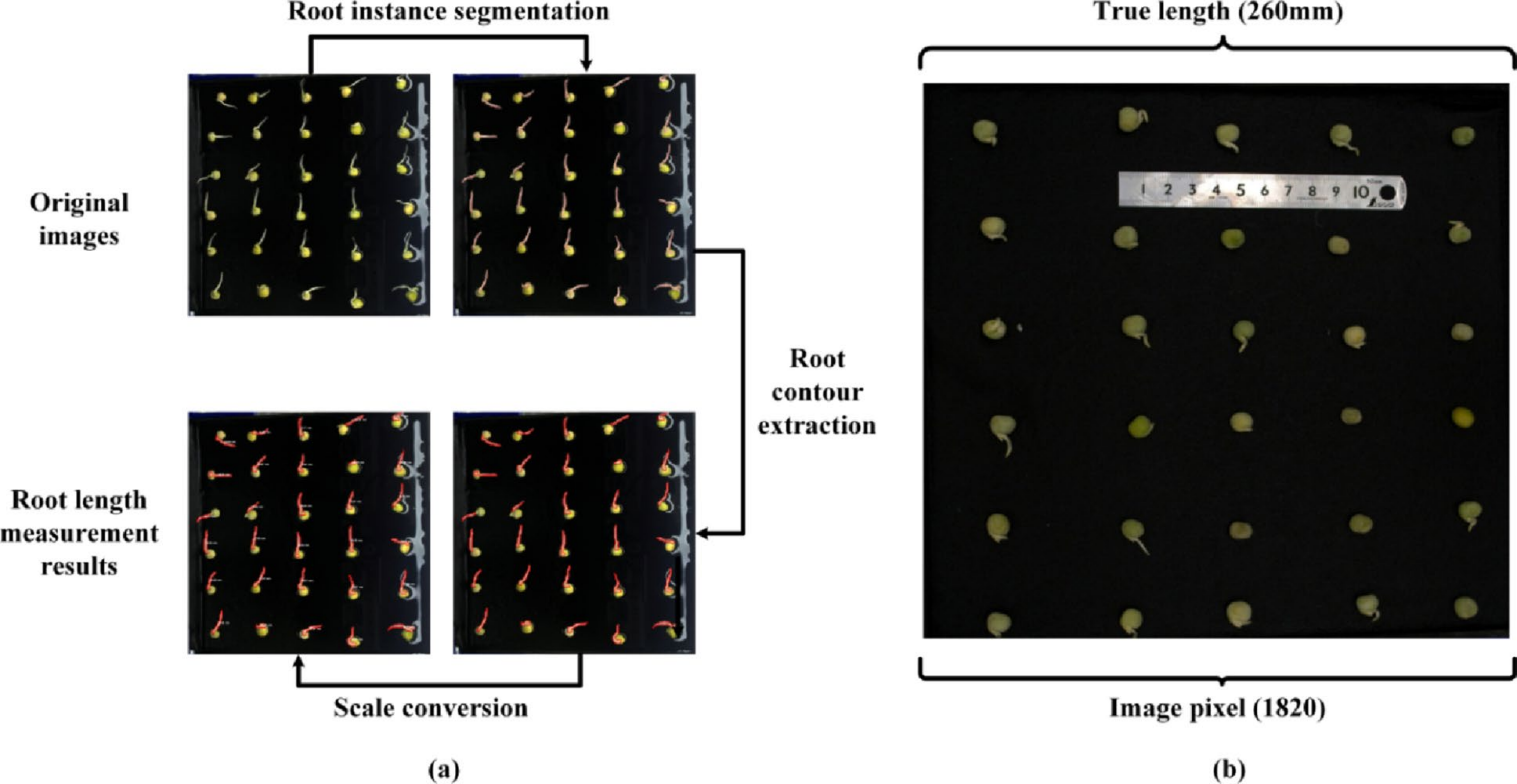
Flowchart of root length calculation

The trained instance segmentation model can effectively locate and segment each pea root from a complex background. However, the model’s output does not directly provide critical biological feature parameters, such as root length. Therefore, a series of refined postprocessing procedure were developed to achieve precise root length measurement. First, contour extraction techniques were used to delineate the boundaries of the pea roots accurately, enabling the calculation of pixel points along the root perimeter. Subsequently, a scale conversion method is applied to standardize the image dimensions, aligning pixel measurements with physical dimensions. Finally, the root length of each pea is obtained by calculating the perimeter pixel points of each pea root. The root length calculation process is illustrated in Fig. 10a.

1. Contour Extraction and Perimeter Calculation: Contours are curves that connect all continuous boundary pixels of the pea root. Given the consistency in color or intensity along the pea root boundaries, this study employs the FindContours algorithm from the OpenCV library [66] to detect contours in the root region from the instance segmentation mask results. By identifying and tracking pixel points along closed curves, the edges of pea roots were accurately defined.
2. Scale Conversion: A set of images was captured with a steel ruler placed on filter paper, as shown in Fig. 10b, to establish the correspondence between the number of pixels in an image and the actual physical dimensions. By precisely calculating the number of pixels corresponding to each millimeter on the scale (denoted as *r*), the relationship between the image pixels and real-world dimensions was established. In this experiment, 1 mm was determined to correspond to seven pixels in the collected images.
3. Root Length Calculation: The formula for calculating pea root length ($\:L$) is provided in Eqs. 10–12. Suppose that the segmentation result of the current pea root is represented by $\:n$ points used for mask segmentation, calculated in a clockwise direction, with each segmented point represented sequentially as ($\:{k}_{1}$, $\:{k}_{2}$, $\:{k}_{3}$……$\:{k}_{n}$). The Euclidean distance between the coordinates of the $\:m$-th annotated point $\:{k}_{m}({\text{X}}_{\text{m}}^{\text{p}},{\:\text{Y}}_{\text{m}}^{\text{p}})$ and the previous point $\:{k}_{m-1}({\text{X}}_{\text{m}-1}^{\text{p}},{\:\text{Y}}_{\text{m}-1}^{\text{p}})$, denoted as (|$\:{k}_{m}$, $\:{k}_{m-1}$|), is calculated and accumulated to acquire the total number of pixels along the contour of the pea root ($\:{S}_{p}$). By dividing the number of contour pixels by *r*, the root perimeter ($\:{S}_{r}$) is obtained. In this study, considering the curvature of the pea root and other factors, half of the perimeter of the segmented root region surrounding the seed was regarded as the predicted length ($\:L$) of the current pea root by the model.

10 $$\:{S}_{p}=\sum\:_{m=1}^{n}{|k}_{m},\:\:{k}_{m-1}|,$$

11 $${S}_{r}={S}_{p}/r,$$

12 $$L=\:{S}_{r\:}/2.$$

### Evaluation metrics of the model for the measurement of pea root length

The following metrics were selected to evaluate comprehensively the performance of the model for pea root length calculation, considering accuracy and the need for a lightweight design: Precision, Recall, average precision ($\:AP$), mean average precision ($\:mAP@0.5$), computational cost (floating point operations [FLOPs]), parameters (Params), and weight file size. The formulas for $\:IoU,\:Precision$,$\:\:Recall$,$\:\:AP$, and $\:mAP@0.5$ are presented in Eqs. 13–17.

13 $$\:IoU=\frac{{P}_{mask}\:\cap\:\:{G}_{mask}}{{P}_{mask}\:\cup\:{\:G}_{mask}},$$

14 $$\:Precision=\frac{TP}{TP+FP},$$

15 $$\:Recall=\frac{TP}{TP+FN},$$

16 $$\:AP={\int\:}_{0}^{1}\:P\left(R\right)dR,$$

17 $$\:\:mAP@0.5=\frac{{\sum\:}_{q=1}^{Q}\:AP\left(q\right)}{Q}.$$

In instance segmentation tasks, the model’s performance evaluation primarily relies on the Intersection over Union ($\:IoU$) between the predicted mask ($\:{P}_{mask}$) and ground truth mask ($\:{G}_{mask}$). $\:IoU$ is the ratio of correctly labeled pixels in the predicted region (segmentation region) to the total pixels in the predicted and actual regions (annotation region). In this study, the $\:IoU$ threshold is set to 0.5. When $\:IoU$ exceeds the threshold, the prediction is considered a successful localization of the target object.

On this basis, true positive ($\:TP$) and false positive ($\:FP$) indicate the number of targets that is correctly and incorrectly classified by the model, respectively. Conversely, if the predicted region has an $\:IoU$ lower than 0.5 when compared with the actual region, it is considered as a missed detection, with false negative ($\:FN$) reflecting the number of missed targets. $\:Precision$ refers to the proportion of correctly identified targets among all predicted targets, whereas $\:Recall$ refers to the proportion of correctly identified targets among all actual targets. On the basis of these two metrics, Precision–Recall curves were plotted for each category, with the area under the curve representing the category’s $\:AP$ value. An $\:AP$ value close to 1 indicates that the model has good performance. The $\:mAP@0.5$ metric is a standardized performance evaluation indicator for instance segmentation tasks, representing the mean of average precision ($\:AP$) across multiple categories($\:Q$). This metric plays an important role in model selection, tuning, and performance comparison.

18 $$\:FLOPs\:\left(Conv\right)=2\times\:H\times\:W\left({C}_{in}{K}^{2}+1\right){C}_{\text{out}},$$

19 $$\:FLOPs\:\left(Pool\right)=\frac{H}{S}\times\:\frac{W}{\text{S}}{\times\:C}_{\text{out}}\times\:K^{2},$$

20 $$\:FLOPs\:\left(FC\right)=2\times{\text{n}}_{\text{i}\text{n}}\times\:{\text{n}}_{\text{o}\text{u}\text{t}}+\:{\text{n}}_{\text{o}\text{u}\text{t}},$$

21 $$\:Params\:\left(Conv\right)={C}_{\text{in}}\times\:{K}^{2}\times\:{C}_{\text{out}\text{}},$$

22 $$\:Params\:\left(FC\right)=({\text{n}}_{\text{i}\text{n}}\times\:{\text{n}}_{\text{o}\text{u}\text{t}}){\:+\text{n}}_{\text{o}\text{u}\text{t}},$$

23 $${\text {Weight File Size}}=\frac{\:Total\:Params\:\times\:\:4}{{1024}^{2}},$$

 

24 $${\text{FPS}}\:=\frac{1000}{\text{T}\text{o}\text{t}\text{a}\text{l}\:\text{i}\text{n}\text{f}\text{e}\text{r}\text{e}\text{n}\text{c}\text{e}\:\text{t}\text{i}\text{m}\text{e}\left(\text{m}\text{s}\right)}.\:\:\:\:\:\:\:\:\:\:\:$$

The computational complexity of the model can be reflected through $\:FLOPs$, whilst the lightweight nature of the model is assessed by the number of *Params*. The weight file size is used to assess whether the model is easy to deploy, and frames per second (FPS) measures the real-time performance of the model during detection. The FLOPs and Params of convolution ($\:Conv$), pooling ($\:Pool$) and fully connected layer ($\:FC$) operations are shown in Eqs. 18–24. $\:H$ere, $\:H$ and $\:W$ represent the height and width of the feature map, respectively; $\:{C}_{in}$ and $\:{C}_{\text{out}}$ denote the input and output channel counts, respectively; $\:K$ represents the kernel size; $\:\text{S}$ indicates the stride. $\:{\text{n}}_{\text{i}\text{n}}$ and $\:{\text{n}}_{\text{o}\text{u}\text{t}}$ are the number of input and output nodes, respectively. $\:Total\:Params$ represents the total parameter count of the model, and $\:\text{T}\text{o}\text{t}\text{a}\text{l}\:\text{i}\text{n}\text{f}\text{e}\text{r}\text{e}\text{n}\text{c}\text{e}\:\text{t}\text{i}\text{m}\text{e}$ = preprocess time + inference time + postprocess time.

## Results

### Training environment and hyperparameter settings

The configuration of the experimental environment is shown in Table 2. The operating system used was Windows 11, with an Intel(R) Xeon(R) Gold 6248R @ 3.00 GHz processor and an NVIDIA GeForce RTX3090 GPU. The deep learning model framework used was PyTorch 2.0.0 [67], and the development language was Python 3.8, with CUDA version 11.7.

**Table 2:** Training environment of the lightweight ARM model

Configuration name	Configuration description
Operating system	Windows 11
Processor	Intel(R) Xeon(R) Gold 6248R @ 3.00 GHz
Graphics processing unit	NVIDIA GeForce RTX 3090
Development language	Python 3.8 + CUDA 11.7
Deep learning framework	PyTorch 2.0.0

None of the models in the ablation and comparison experiments used any pretrained weights during training to ensure the fairness and comparability of the model’s performance. The input image size was adjusted to 640 × 640 pixels, and the number of epochs was set to 100. The key hyperparameter settings during training are shown in Table 3.

**Table 3:** Hyperparameter settings for model training

Hyperparameter name	Setting
Epoch	100
Batch size	8
Image size	640 × 640
Initial learning rate	1 × 10^− 2^
Final learning rate	1 × 10^− 4^
Weight-decay	5 × 10^− 4^
Warmup-epochs	3

### Ablation experiment

In this study, ablation experiments were conducted to analyse the effectiveness of feature distillation and structured pruning techniques. The experimental results are shown in Table 4. In the feature distillation experiment, the teacher model is chosen as YOLOv8-Seg-l, which has a complex structure and a rich number of parameters, to fully capture and learn the feature representations of pea roots. The student model is selected as YOLOv8-Seg-n, which is smaller in size and faster in inference, thereby reducing the model’s demand for computational resources. This allowed the student model to maintain high accuracy while benefiting from a smaller size. Under conventional training conditions, YOLOv8-Seg-n achieved an mAP@0.5 of 89.3%, with strong segmentation performance for the seed part (AP_seed_ reaching 99.3%) but relatively poor performance for root segmentation, wherein its average precision (AP_root_) was only 79.4%. After applying knowledge distillation, mAP@0.5 and AP_root_ increased to 90.6% and 81.7%, respectively, demonstrating the effectiveness of using certain features from the teacher model as auxiliary labels to enhance the student model’s learning performance. This process does not introduce additional parameters to the model, thereby not affecting the inference speed (with the FPS remaining at 62.2).

Furthermore, after implementing structured pruning, the model eliminated a significant number of redundant parameters, resulting in reductions of 44.5% in Params, 31.5% in FLOPs, and 38.3% in the size of the weight files. AP_seed_ remained unchanged, whereas AP_root_ showed only a slight decrease of 0.5%, with FPS increasing to 70.4. These results indicate that structured pruning effectively reduces redundant parameters in the model, improving computational efficiency and inference speed.

**Table 4:** Feature distillation and structured pruning validity analysis

Model	mAP@0.5 (%)	AP_seed_ (%)	AP_root_ (%)	Params (M)	FLOPs (G)	Weight File (MB)	FPS
YOLOv8-Seg-n	89.3	99.3	79.4	3.26	12.1	6.8	62.2
+Knowledge distillation	90.6	99.4	81.7	3.26	12.1	6.8	62.2
+Structured pruning	90.3	99.4	81.2	1.81	8.3	4.2	70.4

This study further explored the effect of feature layer selection on distillation effectiveness in feature distillation, and the results are presented in Table 5.

**Table 5:** Experimental results of different characteristic distillation layers

Feature distillation layer	mAP@0.5(%)	AP_seed_ (%)	AP_root_ (%)	Precision (%)	Recall (%)
None	89.3	99.3	79.4	84.6	71.0
15, 18, 21	90.2	99.4	81.0	83.5	75.5
6, 8, 12, 15, 18, 21	90.6	99.4	81.7	85.0	75.2
2, 4, 6, 8, 12, 15, 18, 21	90.5	99.4	81.5	85.0	74.7

When using the feature vectors from the 15th, 18th, and 21st layers as pseudolabels for distillation, the model’s mAP@0.5, AP_seed_, and AP_root_ metrics improved to 90.2%, 99.4%, and 81.0%, respectively, representing increases of 0.9%, 0.1%, and 1.6% compared with the same metrics of the baseline model (without feature distillation). When six distillation layers (the 6th, 8th, 12th, 15th, 18th, and 21 st layers) were used, the model’s mAP@0.5 and AP_root_ metrics further improved to 90.6% and 81.7%, respectively, with precision and recall reaching 85.0% and 75.2%, respectively. However, when the number of distillation layers increased to eight (the second, fourth, sixth, eighth, 12th, 15th, 18th, and 21 st layers), the model’s mAP@0.5 dropped to 90.5%, and the AP_root_ metric decreased to 81.5%. This finding indicates that selecting an appropriate number and specific distillation layers is crucial for optimising the feature distillation effect.

This study applied multiple iterations of pruning to the distillation-processed YOLOv8-Seg-n model to enhance the effectiveness of the structured pruning strategy. A small pruning ratio (~ 10%) was used during each iteration to refine the model structure progressively. After each pruning round, the model underwent 20 epochs of training on the training set images to fine-tune parameters. As shown in Table 6, the experimental results across different pruning iterations demonstrate that with reduced iterations (e.g., one or two rounds), the model achieved slight performance improvements while reducing the number of parameters. After four iterations, Params, FLOPs, and weight file size reduced by 37.7%, 27.3%, and 31.0%, respectively, with mAP@0.5 slightly decreasing to 90.2%. Following the fifth pruning iteration, the mAP@0.5 and AP_root_ metrics rebounded to 90.3% and 81.2%, whereas Params, FLOPs, and weight file size further reduced to 55.5%, 68.5%, and 61.7% of the same metrics of the original model, respectively. The model, refined through five rounds of pruning, achieves an effective balance between accuracy and lightweight characteristics, making it the optimal choice.

**Table 6:** Experimental results of the effect of the number of iterations on pruning performance

Number of Iterations	mAP@0.5 (%)	AP_seed_ (%)	AP_root_ (%)	Params (M)	FLOPs (G)	Weight File (MB)
0	90.6	99.4	81.7	3.26	12.1	6.8
1	90.8	99.4	82.2	2.89	11.0	6.2
2	90.7	99.4	82.1	2.55	10.2	5.7
3	90.4	99.4	81.5	2.28	9.5	5.2
4	90.2	99.4	81.0	2.03	8.8	4.7
5	90.3	99.4	81.2	1.81	8.3	4.2

### Comparative experiments

The performance metrics of the ARM model were compared in detail with those of other versions in the YOLOv8-Seg series to evaluate the performance of the ARM model for instance segmentation comprehensively. The comparison results are shown in Table 7. This study found that the ARM model had a significantly smaller size than other instance segmentation models in the YOLOv8-Seg series, demonstrating superior lightweight characteristics. Its Params, FLOPs, and weight file size were 1.81 M, 8.3 G, and 4.2 MB, respectively, with an inference speed of 70.4 FPS. Although the root segmentation average precision (AP_root_) of the ARM model was slightly lower than those of the YOLOv8-Seg-l and YOLOv8-Seg-m models, it had improved by 1.0% over that of the YOLOv8-Seg-s model, reaching 81.2%, which sufficiently meets the segmentation needs for the pea root region. Furthermore, compared with YOLOv8-Seg-s, ARM only required 15.3%, 19.4%, and 17.6% of the Params, FLOPs, and weight file size, respectively, significantly improving parameter utilisation efficiency and greatly reducing model size. The optimisation strategies and parameter selections in this study effectively reduced the model’s reliance on computational resources, improving computational efficiency. This improvement is particularly important for applications in resource-constrained environments.

**Table 7:** Experimental results of various evaluation metrics for different models of YOLOv8-Seg instance segmentation

Model	mAP@0.5 (%)	AP_seed_ (%)	AP_root_ (%)	Params (M)	FLOPs (G)	Weight File (MB)	FPS
YOLOv8-Seg-n	89.3	99.3	79.4	3.26	12.1	6.8	62.2
YOLOv8-Seg-s	89.8	99.4	80.2	11.79	42.7	23.8	42.1
YOLOv8-Seg-m	91.1	99.4	82.7	27.24	110.4	54.8	30.7
YOLOv8-Seg-l	91.5	99.4	83.6	45.93	220.8	92.3	23.3
ARM	90.3	99.4	81.2	1.81	8.3	4.2	70.4

In addition, this study compares the performance of the ARM and YOLO series instance segmentation models, with the results shown in Table 7. The results are analyzed from the following two aspects:

1. The reasonableness of choosing YOLOv8-Seg-n as the baseline To address the instance segmentation problem in complex backgrounds and dense objects, the YOLO series models have progressively iterated their versions by incorporating deeper network architectures, more efficient attention mechanisms, and more refined multi-scale feature fusion strategies. However, the core principle of instance segmentation has remained consistent, which is to achieve instance-level segmentation through the combination of bounding boxes and pixel-level masks (referencing YOLACT [68]). Meanwhile, studies [69–72] indicate that with the release of YOLOv5, model performance has stabilized, and version selection in practice now emphasizes compatibility with specific downstream tasks to meet varying accuracy and efficiency requirements across applications. As shown in the Table 8, for the pea root instance segmentation task, the AP_root_ value of the YOLOv8-Seg-n model is improved by 1.0%, 1.2%, 0.9%, and 2.2% compared to YOLOv5-Seg-n, YOLOv10-Seg-n, YOLOv11-Seg, and YOLOv12-Seg, respectively. Meanwhile, the computational cost decreased by 39.2% and 70.7% compared to YOLOv5-Seg-n and YOLOv9-Seg-s, respectively. This result indicates that YOLOv8-Seg-n strikes a good balance between accuracy and lightweight design, validating its reasonableness as a baseline model.
2. The Effectiveness of the Optimisation Strategy: Based on this, the ARM model, constructed using feature distillation and structured pruning techniques, outperforms all models in the YOLO series across various performance metrics, fully proving the effectiveness of the proposed method in this study.

**Table 8:** Experimental results of evaluation metrics for different versions of YOLO instance segmentation models

Model	mAP@0.5 (%)	AP_seed_ (%)	AP_root_ (%)	Params (M)	FLOPs (G)	Weight File (MB)	FPS
YOLOv5-Seg-n [36]	88.9	99.4	78.4	2.96	19.9	5.9	60.5
YOLOv8-Seg-n [38]	89.3	99.3	79.4	3.26	12.1	6.8	62.2
YOLOv9-Seg-s [39]	90.0	99.4	80.6	7.85	41.3	15.6	42.7
YOLOv10-Seg-n [40]	88.8	99.4	78.2	2.72	19.4	5.5	63.1
YOLOv11-Seg [41]	88.9	99.4	78.5	2.84	10.4	5.7	63.7
YOLOv12-Seg [42]	88.3	99.3	77.2	2.82	10.4	5.8	50.2
ARM	90.3	99.4	81.2	1.81	8.3	4.2	70.4

**Fig. 11:**
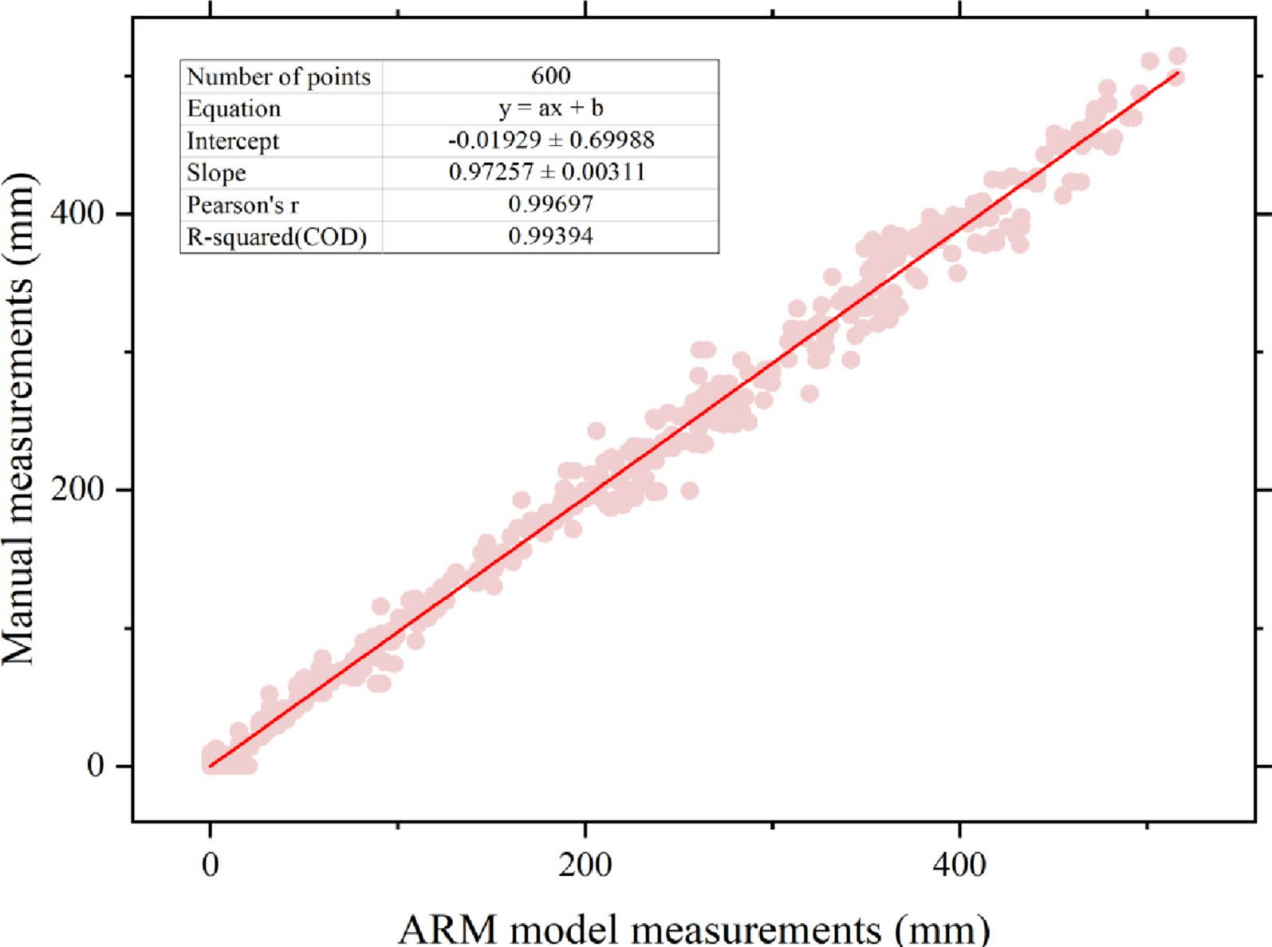
Comparison of fitting results between manual measurements and model-based measurements

Twenty pea germination images were randomly selected from the test set, and 600 root contours were manually annotated to calculate root lengths using pixel conversion. The measurement from the ARM model were then compared with the manual results (Fig. 11). The results showed that the measurements from the ARM model were highly consistent with the manual measurements, with an R² value of 0.993.

### Prediction performance

**Fig. 12:**
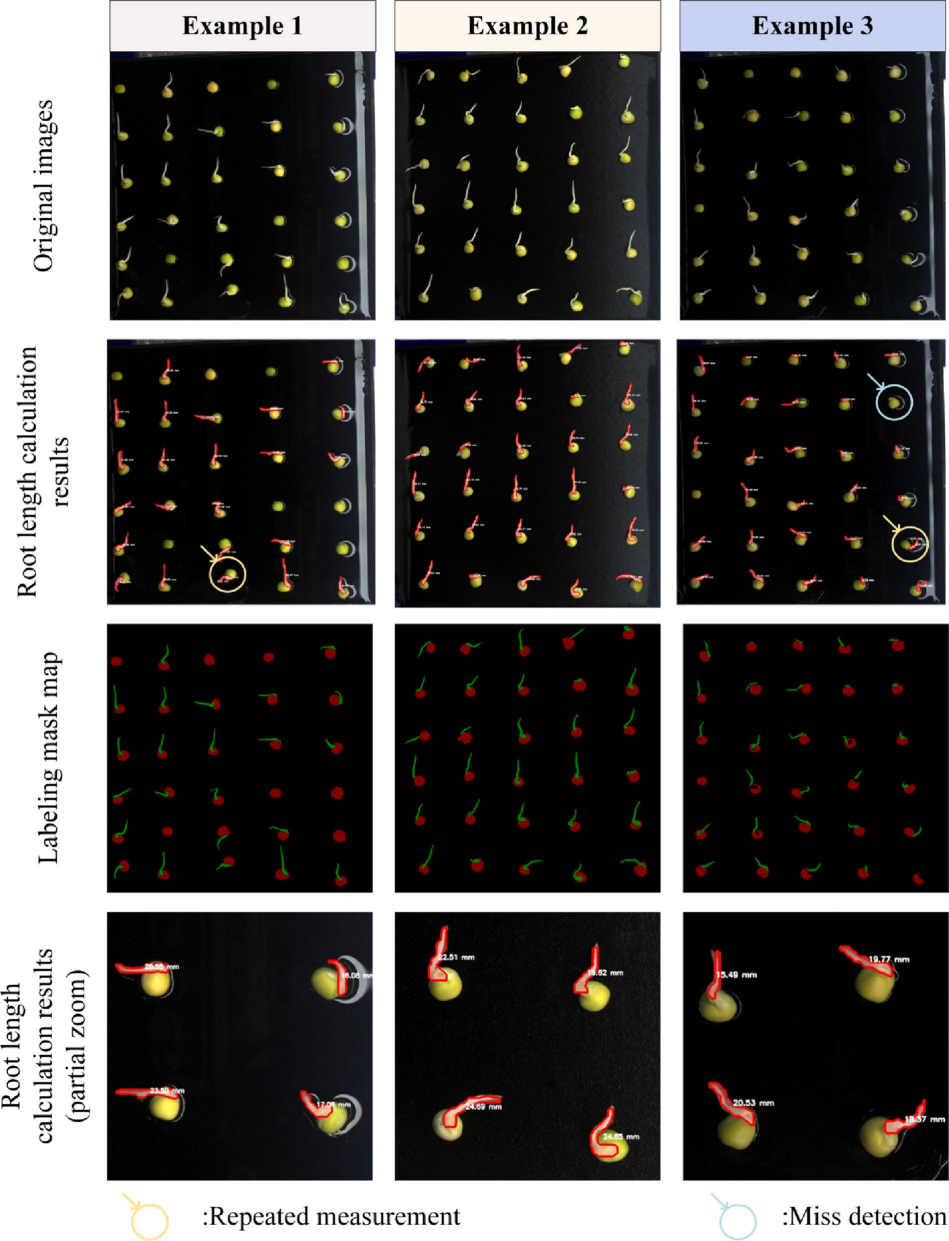
Experimental results of root length calculation by the ARM model

The ARM model developed in this study was specifically designed for root instance segmentation and root length calculation. The results of its performance are displayed in Fig. 12.

In Fig. 12, the first row displays the original images, the second row shows the results of instance segmentation and root length calculation, and the third row presents the mask annotations of the images. Additionally, the fourth row provides a magnified view of some pea root length measurements to illustrate segmentation outcomes. In the second row of Fig. 12 , areas marked with circles highlight the issues observed during the model’s segmentation process. The blue-circled regions indicate instances wherein the model failed to detect early-stage germinating roots, leading to missed detections. Meanwhile, the yellow-circled areas show segmentation errors caused by factors, such as discontinuous root contours, resulting in multiple measurements for a single root. However, overall, ARM demonstrated a high level of consistency with the annotated results in the root segmentation task, validating its effectiveness in root analysis.

### Pea germination experiment and viability evaluation under drought stress

**Fig. 13:**
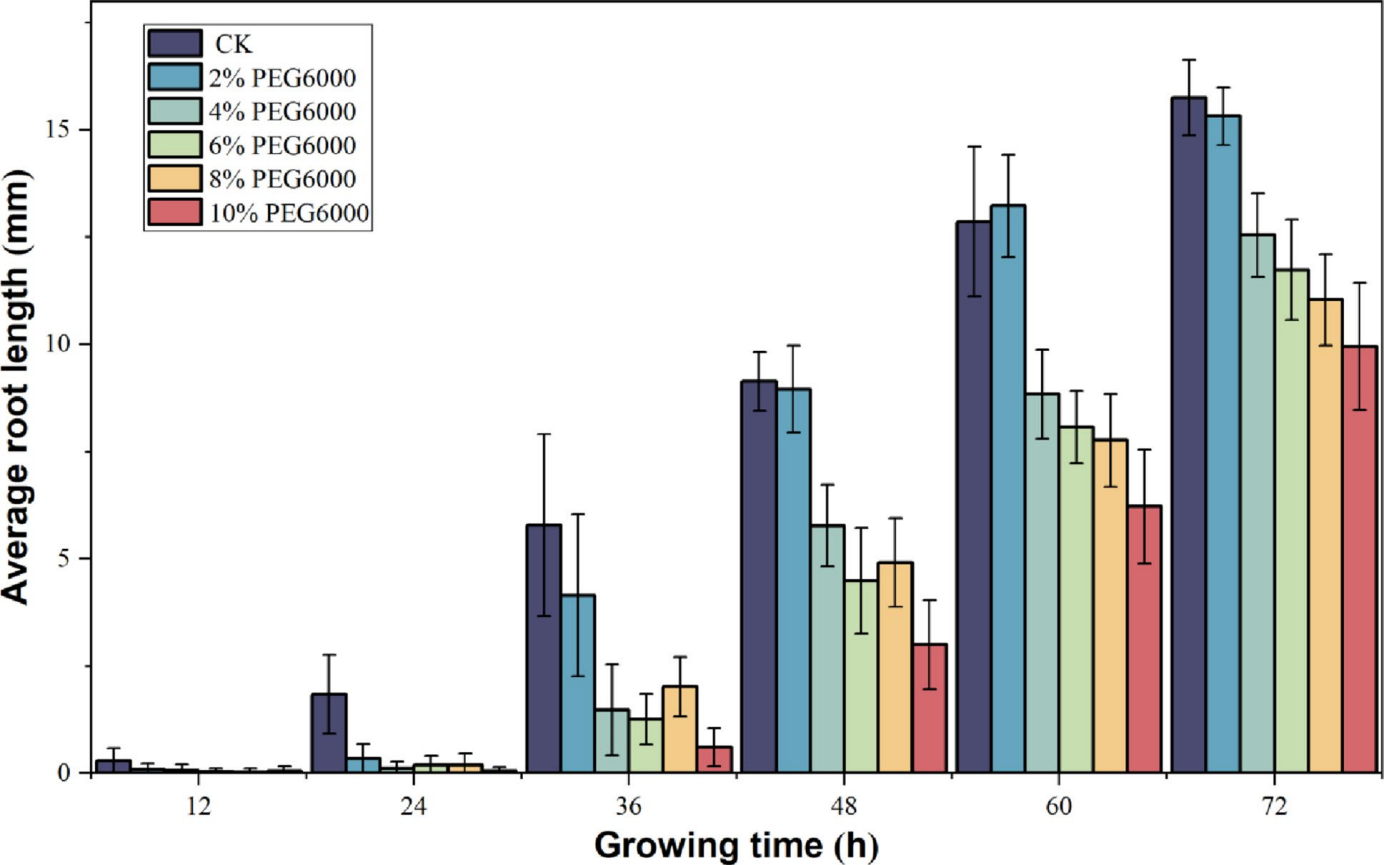
Comparison of average pea root lengths under different drought conditions

**Fig. 14:**
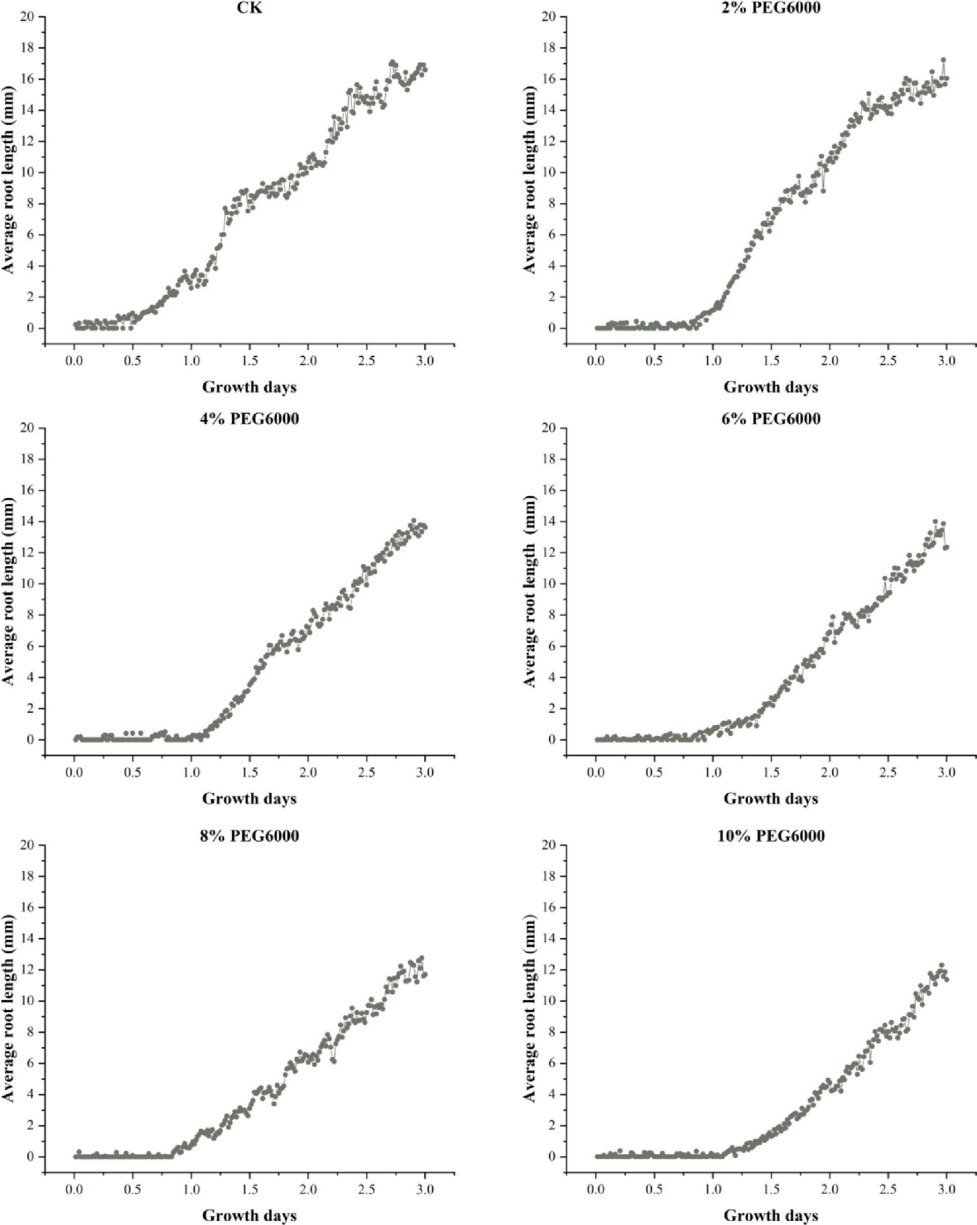
Variation in pea root lengths under different drought conditions

In this study, different drought stress conditions were applied to pea seeds during the seed germination stage, and the developed ARM model was used for full time-series sequence analysis of the changes in root length under varying drought conditions, revealing the crop’s adaptation to drought stress. This provides scientific evidence for the precise breeding of drought-resistant varieties [73]. Given the difficulty of accurately quantifying real-world drought environments, current research mainly utilises polyethylene glycol (PEG-6000) solution, which is non-absorbable and non-toxic to plants. The osmotic pressure generated by this solution inhibits the absorption of water by seeds, thus simulating a drought environment model [74]. Figure 13 presents a quantitative comparison of average root lengths under different drought conditions. Figure 14 illustrates the dynamic trends of root length over time for each treatment, providing a full time-series analysis of root growth processes. As observed, with increasing concentrations of PEG-6000 solution, the root growth rate and length in the later stages of germination showed a gradual decline. In the control group, root growth began between 12 and 24 h, with an average root length of approximately 1.84 mm during this period. By contrast, the treatment groups treated with PEG-6000 solution exhibited no significant root emergence during this time frame, with root growth beginning between 24 and 36 h. In the later stages of germination (60–72 h), the average root lengths in the control and 2% PEG-6000 treatment groups were 15.75 and 15.32 mm, respectively, indicating a mild inhibitory effect. When the concentration of PEG-6000 increased to 4%, the average root length decreased to 12.55 mm. As the concentration rose to 6% and 8%, root growth was further suppressed, with average root lengths being only 11.73 and 11.04 mm, respectively. At a PEG-6000 concentration of 10%, the treatment group exhibited the most significant growth inhibition, with an average root length of only 9.95 mm.

## Discussion

In the task of pea root length measurement, the key to model selection lies in balancing detection accuracy, inference efficiency, and practical deployment feasibility. This study selects the YOLOv8 architecture as the core framework for root length measurement, primarily based on its comprehensive advantages in agricultural scenarios. Compared to two-stage methods like Mask R-CNN, YOLOv8 eliminates redundant computational steps through its single-stage end-to-end prediction and achieves a good balance between accuracy and speed, integrating cross-stage partial networks, multi-scale feature fusion, and dynamic loss optimisation.Many researchers are currently comparing the performance of YOLOv8 with other mainstream instance segmentation models, such as Mask R-CNN, in tasks like apple branch segmentation [75], tomato detection [76], and weed detection [77. The results demonstrate that YOLOv8 consistently outperforms other models in terms of detection accuracy and computational efficiency across various agricultural applications, highlighting its potential advantages in agricultural intelligent perception tasks. 

Moreover, with the continuous updates of the YOLO series, subsequent versions have gradually been introduced into agricultural research, demonstrating good application performance across different crops and environmental conditions. For example, in cross-crop detection, Lu et al. [45] used YOLOv9 for weed and crop recognition, improving cross-domain generalisation and inference speed by adjusting downsampling strategies and lightweight network structures to reduce spatial information loss. Liang et al. [46] employed YOLOv9 for soybean seedling and weed discrimination, combining Mosaic-Dense data augmentation and an improved prediction head to effectively mitigate issues related to small size, similarity, and occlusion. In pest and disease detection, Yuan et al. [47] utilized YOLOv10 for agricultural pest detection, enhancing the detection of small or damaged pests while reducing false positive rates through optimized feature extraction and regression modules. Xiao et al. [48] applied YOLOv11 in remote sensing crop detection, incorporating contextual anchor point attention, multi-scale fusion, and bidirectional feature enhancement to improve small-object detection performance in complex scenarios. Teng et al. [49] used YOLOv11 for rice disease detection, integrating enhanced attention mechanisms, SPPF modules, and a lightweight detection head to achieve efficient, accurate, and cost-effective disease recognition. Eliwa et al. [50] employed YOLOv11 for multi-crop disease classification, designing a customized classification head and dynamic validation mechanism, achieving high-precision multi-disease detection through multi-metric evaluation. Zhang et al. [51] applied YOLOv11 for peanut leaf spot detection and severity quantification, utilizing multi-scale attention and MPDIoU loss to achieve lightweight, real-time high-precision detection under resource-constrained conditions. Yang et al. [52] proposed the GDR-Conv and GTDR-C3 modules based on YOLOv12 and introduced a task-dependent attention mechanism, combining it with the Lookahead optimizer to effectively improve detection of dense, small, and low-contrast weeds while reducing computational costs. Lastly, Sapkota et al. [53] employed YOLOv12 with synthetic images generated by large language models for apple detection, surpassing YOLOv11 and YOLOv10 in performance while significantly reducing data collection costs.

In comparisons between YOLOv8 and other YOLO series models (v9/v10/v11/v12), several official Ultralytics documents [78–80] highlight that YOLOv8 offers significant advantages over YOLOv9, YOLOv10, and YOLOv11 in terms of ecosystem maturity, task diversity (including detection, segmentation, pose estimation, and classification), ease of use, and deployment pipeline integrity. Therefore, in scenarios where project requirements emphasize deployability and end-to-end efficiency, YOLOv8 is often considered a more reliable choice. In specific application studies, Sapkota et al. [81] compared YOLOv8 and YOLOv11 for immature green fruit segmentation in complex orchard environments (including both occluded and non-occluded scenarios). The results showed that while YOLOv11 achieved superior accuracy in several scenarios, YOLOv8 demonstrated the fastest inference speed, at just 3.3 ms. In weed segmentation tasks, Allmendinger et al. [82] conducted a systematic evaluation of YOLOv8, YOLOv9, YOLOv10, and RT-DETR, finding that while accuracy differences among the models were relatively small, YOLOv8n and YOLOv9t excelled in single-frame inference speed. Furthermore, Goyal et al. [83] compared YOLOv8 and YOLOv9 in a potato field weed detection task, where both models exhibited strong classification ability in dense, complex environments. However, YOLOv9 outperformed YOLOv8 in Recall (63.4%), mAP@50 (67.5%), and mAP@50–95 (45.6%), reflecting higher detection and localization accuracy. Notably, YOLOv8 achieved higher precision (70.5%), significantly reducing the false positive rate, thus demonstrating greater robustness in specific applications. In summary, model selection should focus on its adaptability to specific scenarios for more effective extraction of crop features. Additionally, the lightweight root length measurement module proposed in this paper is transferable, and if a more suitable backbone model than YOLOv8 emerges in the future, it can be seamlessly integrated into the current framework to further enhance detection and measurement performance.

It is important to note that in recent years, Transformer-based models (such as the DETR series) have shown potential to surpass YOLO in certain agricultural tasks. However, the decision to select the YOLO series models in this study is primarily based on considerations of model architecture and ecosystem. First, at the architecture level, the agricultural field generally lacks large-scale high-quality data, and annotation costs are high [84]. YOLO leverages convolutional priors and anchor-based/anchor-free mechanisms to effectively learn features under limited sample conditions, with faster convergence compared to Transformer-based DETR models, which often require longer training periods and more data to achieve similar performance [85, 86]. Therefore, in data-scarce agricultural scenarios requiring rapid iteration, YOLO is more adaptable. Second, in terms of ecosystem, the YOLO series has been validated in numerous industrial applications, particularly excelling in small-object detection and complex backgrounds, with a well-established open-source implementation and tool support, facilitating efficient deployment across various hardware environments. In contrast, although Transformer-based methods (e.g., RF-DETR) have shown promise in some tasks, application cases remain limited, and the ecosystem is still evolving, which hinders stability and the ease of model development and deployment. Therefore, when performance differences are not significant, selecting YOLO is a more reliable choice, balancing training efficiency, model performance, and practical feasibility.

In the future, as agricultural image datasets continue to expand and cross-crop, cross-scenario data accumulate, Transformer-based architectures may achieve better performance [87]. Transformers offer stronger scalability and transfer learning capabilities, enabling rapid adaptation to specific tasks in small-sample scenarios, such as root length measurement or disease identification for different crops [88, 89]. Furthermore, Transformer models inherently excel at integrating multimodal and temporal information. In future agricultural research, root measurements will likely rely not only on single-frame images but also on multispectral, 3D imaging, temporal data (e.g., dynamic changes in root systems throughout growth stages), and potentially expert knowledge. The Transformer architecture is well-suited for unified modeling of multimodal inputs, facilitating more comprehensive and robust analysis [90]. In the future, the transfer learning capabilities of Transformer-based models could replace YOLO’s backbone architecture to further enhance model performance in complex, small-sample scenarios.

Moreover, with the advancement of large language models (LLMs), LLM-driven text-to-image generation techniques provide a novel solution to the data scarcity problem. Existing studies have shown that synthetic images generated through diffusion models or LLMs have significantly improved detection accuracy in tasks such as wheat spike detection [91] and apple recognition [92, 93], while effectively reducing data collection and annotation costs. In the future, incorporating synthetic data in pea root length measurement tasks could expand the long-tail sample diversity under various lighting and cultivation conditions, while semi-automated annotation and active learning could further enhance the model’s robustness and scalability.

## Conclusions

This study presents a systematic investigation centered on the dynamic measurement and analysis of pea root length, aiming to provide scientific support for improving pea stress resistance, stabilizing yield, and promoting sustainable agricultural development. By independently developing a full-sequence, high-throughput seed germination image acquisition system, we comprehensively collected imaging data covering the entire germination period of peas and constructed a high-quality pea germination dataset comprising 2,400 images. Building upon this dataset, we developed a lightweight root length measurement model, termed ARM, based on an instance segmentation algorithm, capable of accurately measuring root length during pea germination. The model significantly reduces dependence on storage and computational resources, thereby greatly enhancing both the efficiency of root sample processing and the practical utility of the model, effectively replacing manual measurement methods. Furthermore, continuous monitoring of root length in “Zhonghua No.6” peas under six drought conditions (CK, 2%, 4%, 6%, 8%, and 10% PEG-6000) was conducted to quantitatively analyze differences in root growth trends across various stress treatments, thereby further validating the reliability and effectiveness of the proposed model in real-world scenarios.

## Supplementary Information

Supplementary Material 1.

## Data Availability

Data will be made available on request.

## Abbreviations

AP: Average precision
ARM: Automatic root measurement
BN: Batch normalization
CSP: Cross-Stage Partial Networks
CK: Control check
CNNs: Convolutional Neural Networks
FC: Fully connected layer
FLOPs: Floating point operations
FN: False negative
FP: False positive
FPS: Frames per second
IoU: Intersection over Union
KL: Kullback–Leibler
YOLO: You Only Look Once
