# Supplementary material for: Automatic root measurement: a lightweight method for measuring pea root length
Source: Plant Methods. 2025 Dec 8;21:162. doi: 10.1186/s13007-025-01479-1 (PMC12701599; doi:10.1186/s13007-025-01479-1)
Supplement: Supplementary file 1 — Supplementary Material 1. [file 13007_2025_1479_MOESM1_ESM.docx]

**Supplementary Material**

1. **Summary of existing research on YOLO for agricultural applications**

Supplementary Table 1 Summary of existing research on YOLO for agricultural applications.

| Model | Reference | Research focus | Optimization Objective |
| --- | --- | --- | --- |
| YOLOv8 | Tian et al. [43] | Tomato seed germination vigor phenotyping | Combines micro-object detection and global attention to improve vigor assessment by reducing seed damage, simplifying operation, and enhancing accuracy. |
|  | Sun et al. [44] | Pear pollen phenotypic analysis | Uses data augmentation to improve robustness in complex pollen-root environments. |
| YOLOv9 | Lu et al. [45] | Multi-crop weed/crop detection | Adjusts downsampling and lightens network to reduce spatial loss, improving cross-domain generalization and inference speed. |
|  | Liang et al. [46] | Soybean seedling and weed discrimination | Applies Mosaic-Dense augmentation and refines prediction head to handle small size, similarity, and occlusion between seedlings and weeds. |
| YOLOv10 | Yuan et al. [47] | Agricultural pest detection | Enhances feature extraction and regression to improve small/damaged pest detection while reducing false positives. |
| YOLOv11 | Xiao et al. [48] | Crop detection in remote sensing imagery | Integrates contextual anchor attention, multi-scale fusion, and bidirectional features to boost small-object detection in complex scenes. |
|  | Teng et al. [49] | Rice disease detection | Combines enhanced attention, SPPF module, and lightweight detection head to improve efficiency, accuracy, and cost-effectiveness. |
|  | Eliwa et al. [50] | Multi-crop plant disease classification | Employs custom classification head, dynamic validation, and multi-metric evaluation for high-precision multi-disease detection. |
|  | Zhang et al. [51] | Peanut leaf spot disease detection and severity quantification | Uses multi-scale attention and MPDIoU loss for lightweight, accurate real-time detection of dense small lesions in constrained hardware. |
| YOLOv12 | Yang et al. [52] | Multi-crop weed detection | Introduces GDR-Conv, GTDR-C3 with task-dependent attention, and Lookahead optimizer to lower computation while improving detection of dense, small, low-contrast weeds. |
|  | Sapkota et al. [53] | Apple detection | Trains on LLM-generated synthetic images, surpassing YOLOv11/YOLOv10 in accuracy and reducing data collection costs. |

1. **Growth characteristics of “Zhonghua No. 6” peas**

Supplementary Table 2 Growth characteristics of Zhonghua No. 6 peas

|  | **Parameters** | **Value** |
| --- | --- | --- |
| Growth parameters | Height | 40-50 cm |
|  | Single plant pods | 7-10 |
|  | Pod length | 7-9 cm |
|  | Pod width | 1.0-1.2 cm |
| Growing period | Spring sowing areas | 65-75 days |
|  | Winter cropping areas | 90-100 days |
|  | Overwintering of seedlings | 150-160 days |

The stems and leaves of Zhonghua No. 6 are dark green, and the plant produces white flowers and hard pods. The internodes exhibit a swollen morphology. During the early growth stage, the green pods contribute significantly to the yield, accounting for approximately 50% of the total production. The pods are large and well-filled, which enhances their appeal compared to smaller pods. The dried peas are light green in color, with a hundred-seed weight of approximately 25 grams. This variety demonstrates strong adaptability, excellent cold tolerance, and notable resistance to powdery mildew.

1. **Mean root length of Zhonghua No. 6 peas under different drought conditions**

Supplementary Table3 Mean root length of Zhonghua No. 6 peas under different drought conditions over 0–72 h.

| **Germination times** | **Treatment** | **Root length** |
| --- | --- | --- |
| 0-12h | CK | 0.29±0.29 |
|  | 2% PEG-6000 | 0.09±0.14 |
|  | 4% PEG-6000 | 0.07±0.13 |
|  | 6% PEG-6000 | 0.05±0.08 |
|  | 8% PEG-6000 | 0.03±0.08 |
|  | 10% PEG-6000 | 0.06±0.11 |
| 12-24h | CK | 1.84±0.92 |
|  | 2% PEG-6000 | 0.34±0.34 |
|  | 4% PEG-6000 | 0.11±0.16 |
|  | 6% PEG-6000 | 0.20±0.21 |
|  | 8% PEG-6000 | 0.19±0.27 |
|  | 10% PEG-6000 | 0.05±0.09 |
| 24-36h | CK | 5.78±2.12 |
|  | 2% PEG-6000 | 4.15±1.89 |
|  | 4% PEG-6000 | 1.48±1.06 |
|  | 6% PEG-6000 | 1.26±0.59 |
|  | 8% PEG-6000 | 2.02±0.69 |
|  | 10% PEG-6000 | 0.61±0.44 |
| 36-48h | CK | 9.14±0.69 |
|  | 2% PEG-6000 | 8.95±1.02 |
|  | 4% PEG-6000 | 5.78±0.96 |
|  | 6% PEG-6000 | 4.49±1.24 |
|  | 8% PEG-6000 | 4.92±1.03 |
|  | 10% PEG-6000 | 3.00±1.03 |
| 48-60h | CK | 12.86±1.74 |
|  | 2% PEG-6000 | 13.23±1.19 |
|  | 4% PEG-6000 | 8.83±1.04 |
|  | 6% PEG-6000 | 8.07±0.84 |
|  | 8% PEG-6000 | 7.76±1.08 |
|  | 10% PEG-6000 | 6.22±1.34 |
| 60-72h | CK | 15.75±0.88 |
|  | 2% PEG-6000 | 15.32±0.68 |
|  | 4% PEG-6000 | 12.55±0.97 |
|  | 6% PEG-6000 | 11.73±1.17 |
|  | 8% PEG-6000 | 11.04±1.07 |
|  | 10% PEG-6000 | 9.95±1.48 |

1. **Discussion**

Currently, root length analysis methods based on CNNs, such as RootPainter , RootDetector, and MyROOT, primarily focus on the root analysis of single, complex root system plants. Consequently, most studies employ semantic segmentation techniques. While these techniques offer high accuracy in handling complex scenarios involving intertwined lateral root growth, their limitation is the inability to distinguish between different individuals, typically analyzing only a single plant, which results in lower analysis efficiency. In contrast, this study focuses on analyzing the drought resistance of peas, with a particular emphasis on the root system during the germination stage. During this phase, pea roots have relatively fewer lateral roots, making measurements less challenging compared to more complex root systems such as those in Arabidopsis. Therefore, one of the key objectives of this research is to improve the efficiency of root analysis (measured by the number of seeds present in a single image). Since instance segmentation techniques not only allow for precise segmentation of root regions but also effectively differentiate between individual plants, they are considered the optimal choice for pea root segmentation.

Moreover, current research methods often primarily focus on accuracy while overlooking deployment costs and inference efficiency, both of which are critical factors in determining whether a model can be applied in practical agricultural production. To address this, this study optimizes the YOLOv8-Seg-n model through feature distillation and structured pruning techniques, resulting in the ARM model. This optimization successfully achieves an effective balance between accuracy and deployment cost, meeting the practical needs of agricultural production. Notably, the method presented in this study demonstrates strong generalizability, allowing for effective transfer to different versions of the YOLO series models. As YOLO adapts to downstream tasks, the performance of ARM will continue to improve.

However, the root length measurements in this study may be influenced by two factors, potentially introducing errors: (1) Non-perpendicular orientation between pea roots and the camera: When the growth direction of pea roots forms an angle with the horizontal plane, the projected length in 2D imaging may be shorter than the actual length, leading to underestimation. Although roots during germination predominantly grow horizontally, minor measurement deviations may still occur. (2) Overlapping of Pea Roots: When root systems intersect or overlap, instance segmentation models can distinguish individual root systems; however, in cases with large overlapping areas or complete occlusion of roots, the lack of sufficient visible feature information hinders effective measurement, thereby introducing errors. In the future, incorporating technologies such as three-dimensional modeling, multi-angle imaging, or depth information acquisition could help mitigate the adverse effects caused by viewpoint bias and severe occlusion.
